# A new iguanodontian (Dinosauria: Ornithopoda) from the Early Cretaceous of Mongolia

**DOI:** 10.7717/peerj.5300

**Published:** 2018-08-03

**Authors:** Terry A. Gates, Khishigjav Tsogtbaatar, Lindsay E. Zanno, Tsogtbaatar Chinzorig, Mahito Watabe

**Affiliations:** 1 Department of Biological Sciences, North Carolina State University, Raleigh, NC, USA; 2 Paleontology Unit, North Carolina Museum of Natural Sciences, Raleigh, NC, USA; 3 Department of Paleontology, Institute of Paleontology and Geology, Mongolian Academy of Sciences, Ulaanbataar, Mongolia; 4 Department of Natural History and Earth Sciences, Faculty of Sciences, Hokkaido University, Hokkaido University Museum, Sapporo, Japan; 5 School of International Liberal Studies, Waseda University, Tokyo, Japan

**Keywords:** Dinosaur, Ornithopod, Evolution, Asia, Anatomy, Biodiversity, Phylogeny, Histology, Ontogeny, Paleontology

## Abstract

We describe a new iguanodontian ornithopod, *Choyrodon barsboldi* gen. et sp. nov. from the Albian-aged Khuren Dukh Formation of Mongolia based on several partial skeletons interpreted to represent a subadult growth stage based on osteohistological features. This new taxon is diagnosed by many autapomorphies of the maxilla, nasal, lacrimal, opisthotic, predentary, and surangular. *Choyrodon* displays an unusual combination of traits, possessing an open antorbital fenestra (a primitive ornithopod trait) together with derived features such as a downturned dentary and enlarged narial fenestra. Histological imaging suggests that the type specimen of *Choyrodon* would have been a subadult at the time of death. Phylogenetic analysis of two different character matrices do not posit *Choyrodon* to be the sister taxon or to be more primitive than the iguanodontian *Altirhinus kurzanovi*, which is found in the same formation. The only resolved relationship of this new taxon is that it was hypothesized to be a sister-taxon with the North American species *Eolambia caroljonesa*. Though discovered in the same formation and *Choyrodon* being smaller-bodied than *Altirhinus*, it does not appear that the former species is an ontogimorph of the latter. Differences in morphology and results of the phylogenetic analyses support their distinction although more specimens of both species will allow better refinement of their uniqueness.

## Introduction

The diversity of ornithopod dinosaurs has increased dramatically over the past decade due to new discoveries and reassessment of previous finds (Paul, 2008; Wu, Godefroit & Hu, 2010; McDonald, 2011, 2012a). Accurate understanding of this diversity is essential for deciphering the pattern of skeletal evolution that occurred between basal forms and specialized hadrosaurids. Current evidence suggests that morphological changes in this clade did not occur in a stabilizing, stepwise fashion. Rather, several key derived features, such as the antorbital fenestra, rostral premaxillary expansion, external naris expansion, and multiple dental traits, exhibit a mosaic evolutionary pattern—variably appearing during the early evolution of Iguanodontia from the Early Cretaceous (e.g., *Jinzhousaurus yangi*, Wang & Xu, 2001; Barrett et al., 2009) through the Late Cretaceous (Barrett et al., 2009; McDonald, 2012b). Here, we report a new iguanodontian from the Lower Cretaceous of Mongolia that displays a combination of primitive and derived cranial characteristics.

**Figure 1:**
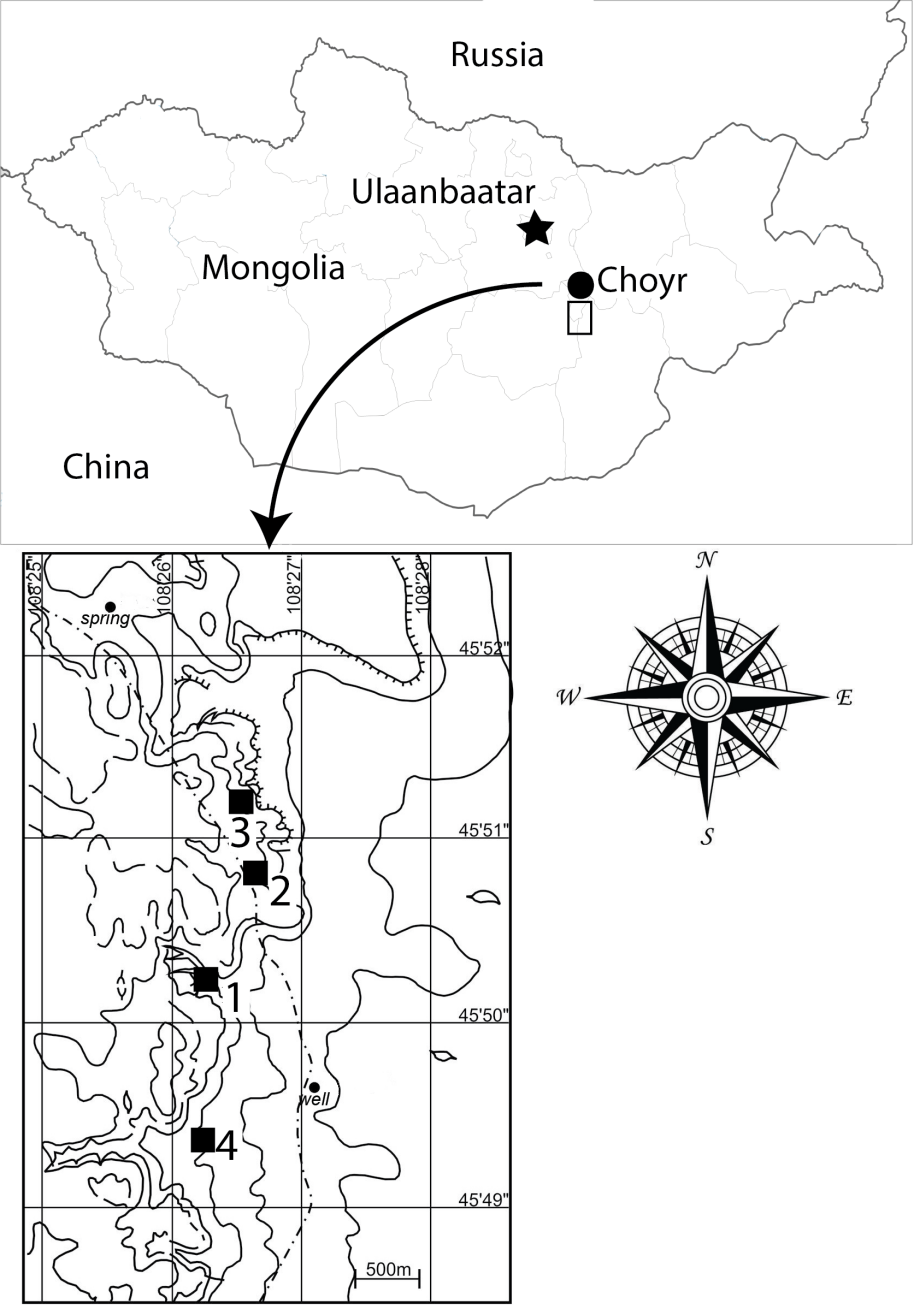
Map of Mongolia showing Khuren Dukh locality. Contour lines demarcate topography around fossil sites of Khuren Dukh. 1-site for *Choyrodon barsboldi* MPC-D100/800; 2-site for *Choyrodon barsboldi* MPC-D100/801; 3-site for *Choyrodon barsboldi* MPC-D100/803; 4-site for *Harpimimus okladnikovi*. Mongolia map credit: Terry Gates. Site location map modified from Watabe et al. (2004) by Khishigjav Tsogtbaatar and Terry Gates.

Specimens of the new taxon were discovered at the Khuren Dukh locality of the Lower Member of the Khuren Dukh Formation (Ito et al., 2006) in southeastern Mongolia (Fig. 1), the same Lower Cretaceous beds that yielded the iguanodontian *Altirhinus kurzanovi* (Norman, 1998), the basal ornithomimosaur *Harpymimus okladnikovi* (Barsbold & Perle, 1984), several species of turtles (Suzuki & Narmandakh, 2004), and isolated fish remains (T. Gates, 2014, personal observation). Hicks et al. (1999) dated the Khuren Dukh locality to Aptian? age based on palynologic evidence, however, the age was later redefined by Nichols, Matsukawa & Ito (2006) as middle to late Albian, also based on palynology.

## Systematic Paleontology

Dinosauria (Owen, 1842)
Ornithischia (Seeley, 1888)
Ornithopoda (Marsh, 1881)
Iguanodontia (Dollo, 1888) (sensu Sereno, 1986) urn:lsid:zoobank.org:act:07C25E64-6C15-40C3-8A61-A6378D8494AD
*Choyrodon* gen. nov. urn:lsid:zoobank.org:act:A11FF4A2-903D-4BE5-84A2-66D19E545A61
*Choyrodon barsboldi* sp. nov. urn:lsid:zoobank.org:act:92591A61-54AD-4E74-B47F-2F1135305E9A.

### Holotype

MPC-D 100/801: A partial disarticulated skull containing right and left premaxillae, right maxilla, right ectopterygoid, right palatine, left pterygoid, right and left jugals, right and left lacrimals, right nasal, nearly complete skull roof (including frontals, a single prefrontal (right?), postorbitals, squamosals, parietals, laterosphenoids, orbitosphenoids, prootics, exoccipitals, opisthotics, and supraoccipital), right and left quadratojugals, right quadrate, right and left dentaries, partial predentary, right and left surangulars, right and left angulars, metacarpals, and cervical ribs.

### Etymology

*Choyrodon*—*Choyr*, a city near the Khuren Dukh locality where this taxon was discovered; *don*—latin, meaning tooth, a common ending for ornithopod dinosaur taxa.

*barsboldi*—named after Dr. Rinchen Barsbold, a leading dinosaur paleontologist of Mongolia and leader of the paleontology expedition that discovered the first remains of this species.

### Locality, horizon, and geologic age

The quarry from which the holotype specimen derives is located within a thick brown–black organic rich siltstone at the Khuren Dukh locality of the Lower Member of the Khuren Dukh Formation (sensu Ito et al., 2006). Approximate age is middle to late Albian (Nichols, Matsukawa & Ito, 2006). Exact locality information is on file at the MPC.

### Referred materials

MPC-D 100/800: A partial disarticulated skull and fragmentary postcranial skeleton containing right and left premaxillae, right and left nasals, right quadrate, quadratojugal (unknown side), nearly complete skull roof missing prefrontals and squamosals, right surangular, partial predentary, left sternal rib, neural spine.

MPC-D 100/803: A fragmentary skull and partial postcranial skeleton with left nasal, left scapula, left coracoid, left radius, left ulna, partial manus, vertebrae, ribs, partial left pubis, left ischium, partial left ilium, right femur, partial left tibia.

### Diagnosis

Iguanodontian ornithopod distinguished by the following autapomorphies (marked with an asterisk) and unique combination of characters: anterodorsal process of maxilla dorsoventrally broad relative to total height of maxilla, with a length to height ratio of 1 and the dorsal margin of this process reaching and extending along a portion of the ventral bony naris; anterodorsal margin of maxilla horizontal*; nasal possessing low rise on dorsal surface positioned at posterior extent of nasal fenestra*; expansion of the distal lateral process of premaxilla; dorsoventrally thickened bone on posterior surface of nasal below dorsal rise; elongate, hypertrophied external narial fenestra with nasals comprising a small portion of posteroventral margin; antorbital fenestra; lacrimal bearing rounded anterior margin and lobate shape in lateral view*; squamosal processes of postorbital deflected posterodorsally; quadrate notch located at midheight of element; posterior process of opisthotic? wraps over to contact broadly the posterior face of supraoccipitals*; predentary with flattened articulation surface across entire ventral margin*; predentary with series of paired foramina below oral margin throughout lateral length of element*; distal end of dentary deflected ventrally; surangular displays two deep osteological folds on the lateral surface ventral to second foramen*; prepubic process of pubis deflects ventrally.

### Remarks

The electronic version of this article in portable document format will represent a published work according to the International Commission on Zoological Nomenclature (ICZN), and hence the new names contained in the electronic version are effectively published under that Code from the electronic edition alone. This published work and the nomenclatural acts it contains have been registered in ZooBank, the online registration system for the ICZN. The ZooBank LSIDs (Life Science Identifiers) can be resolved and the associated information viewed through any standard web browser by appending the LSID to the prefix http://zoobank.org/. The LSID for this publication is: zoobank.org:pub:E44CAFD0-8E9A-4025-80F2-78011BA0594F. The online version of this work is archived and available from the following digital repositories: PeerJ, PubMed Central, and CLOCKSS.

## Description

General comments—At least three partial specimens are known including the holotype specimen MPC-D 100/801 and two referred specimens MPC-D 100/800 and MPC-D 100/803. The skull is completely known, except for the braincase and vomer. Several postcranial skeletal elements were recovered with MPC-D 100/803. MPC-D 100/800 and MPC-D 100/803 are referred to *C. barsboldi* based on autapomorphic characteristics of the nasal. Each of the specimens is nearly identical in size, suggesting an equivalent ontogenetic stage for all referred specimens. We include comparisons to other iguanodontian species to help define the anatomy of *Choyrodon*. Specific reference is made to *Altirhinus* to distinguish *Choyrodon* from this co-occurring species.

**Figure 2:**
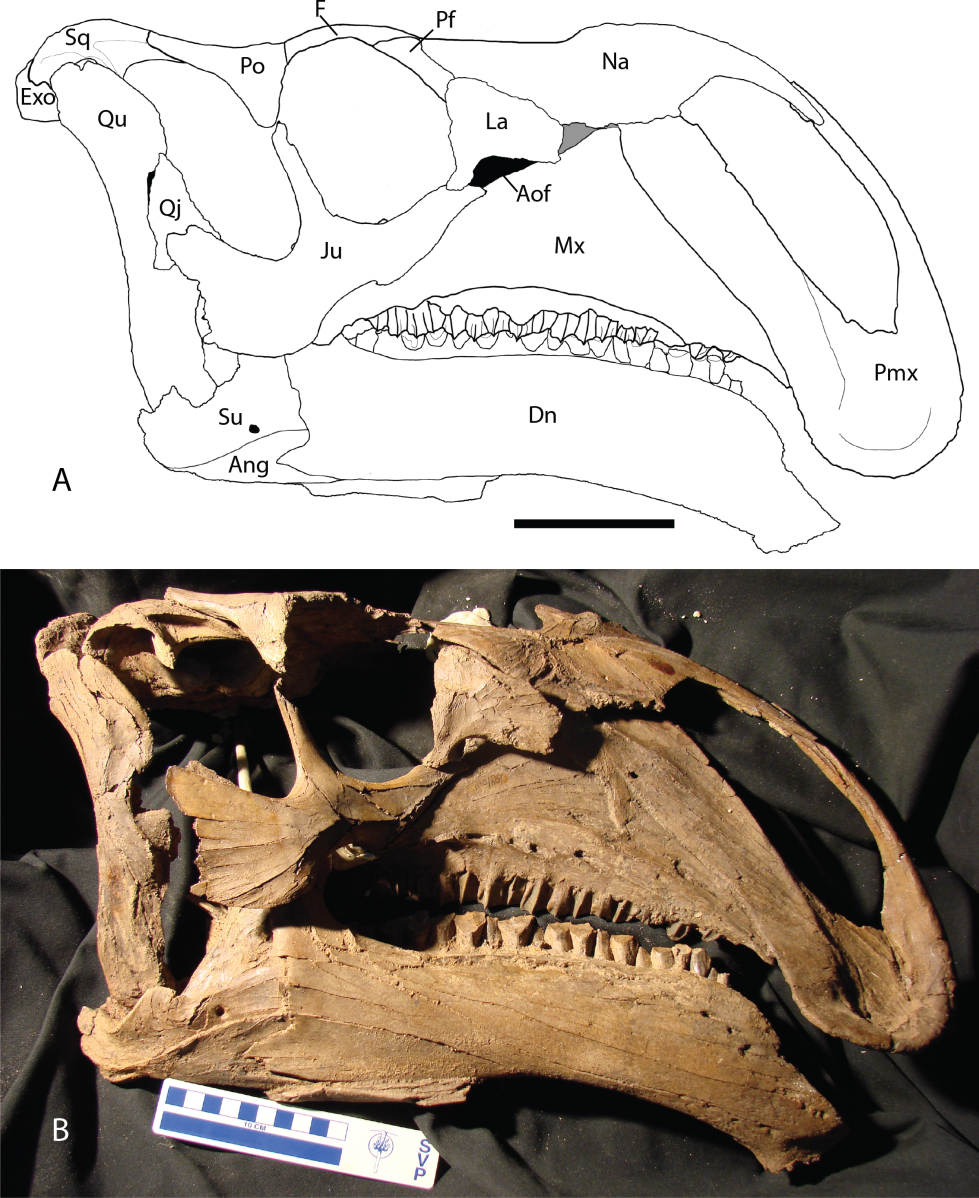
Reconstructed skull of Chyorodon type specimen MPC-D 100/801. (A) Line drawing reconstruction of based on elements present in MPC-D 100/801. The gray filled region near the maxilla, nasal, and lacrimal represents an uncertain relationship of the bones due to incomplete specimens. The frontal–prefrontal contact has been estimated on this figure, as it is not easily seen on the original specimen. (B) Bones of MPC-D reconstructed. Study sites: Ang, angular; Aof, antorbital fenestra; Dn, dentary; Exo, exoccipital; F, frontal; Ju, jugal; La, lacrimal; Mx, maxilla; Na, nasal; Pf, prefrontal; Pmx, premaxilla; Po, postorbital; Qj, quadratojugal; Qu, quadrate; Sq, squamosal; Su, surangular. Scale bar equals 10 cm. Illustration credit and photograph credit: Terry Gates.

**Figure 3:**
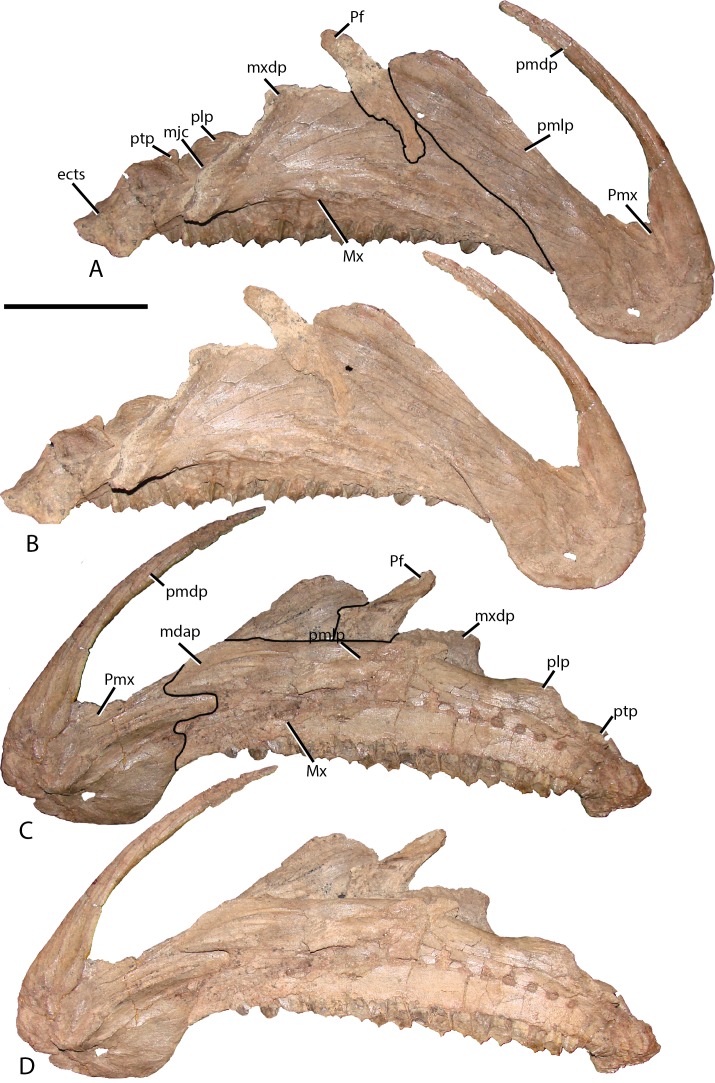
Premaxilla and maxilla of MPC-D 100/801. (A) Lateral view with lines demarcating elements; (B) lateral view without lines; (C) medial views with lines demarcating elements; and (D) medial view without lines. Prefrontal is adhered to the maxilla but does not occur in this position in articulation. Study sites: ects, ectopterygoid shelf; mdap, maxilla dorsal anterior process; mjc, maxilla-jugal contact; Mx, maxilla; mxdp, maxilla dorsal process; Pf, prefrontal; plp, palatine process; pmdp, premaxilla dorsal process; pmlp, premaxilla lateral process; Pmx, premaxilla; ptp, pterygoid process. Scale bar equals 10 cm. Photograph credit: Terry Gates.

**Figure 4:**
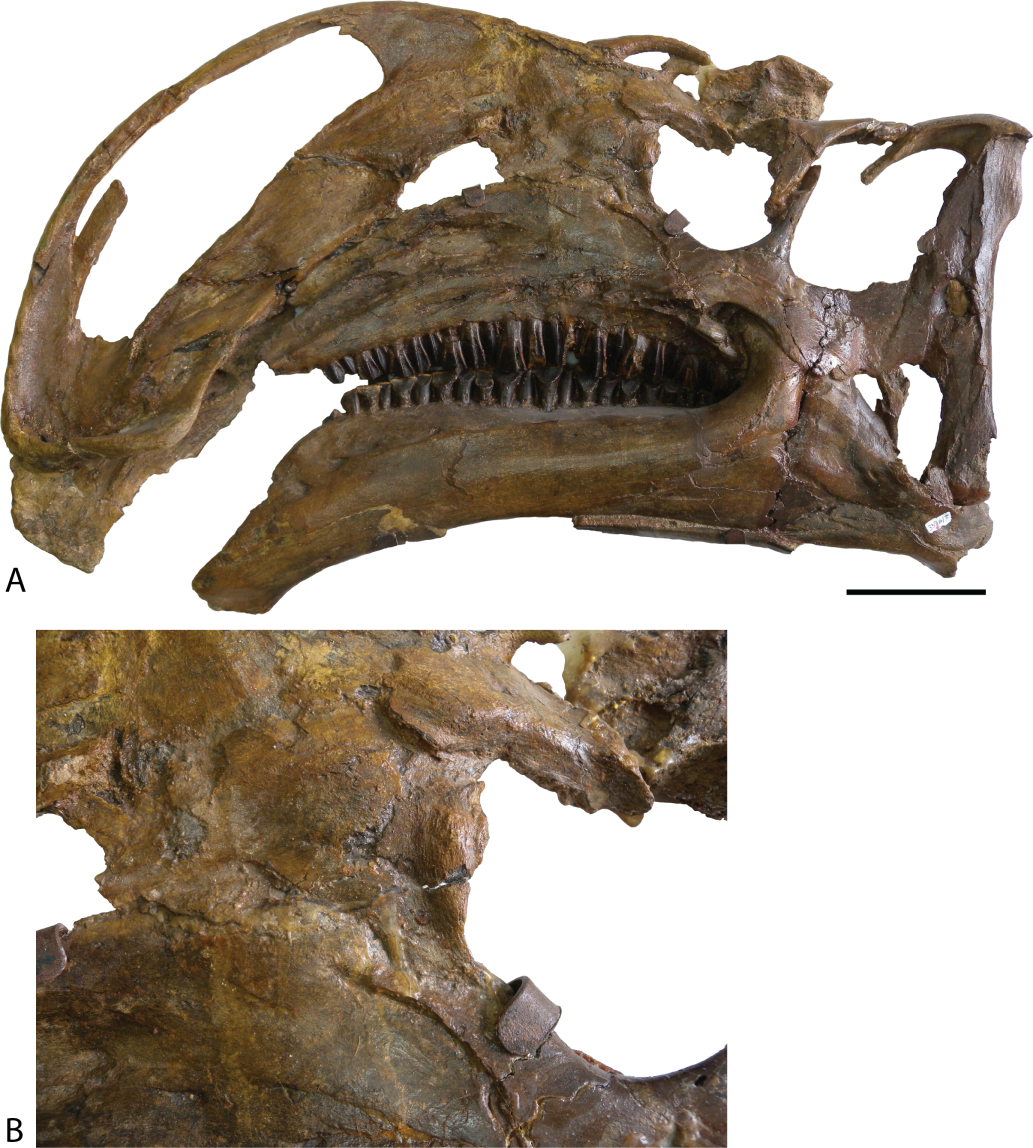
Type specimen of *Altirhinus*. (A) In lateral view; (B) close up of lacrimal and maxilla showing the region that would contains the antorbital fenestra in *Choyrodon*. Scale bar equals 10 cm. Photograph credit: Khishigjav Tsogtbaatar.

Premaxilla—The rostral region of the premaxilla bends ventral to the level of the dentary tooth row (Figs. 2 and 3), a condition shared with *Altirhinus* (Norman, 1998; Fig. 4), *Probactrosaurus gobiensis* (Norman, 2002), *Equijubus normani* (Hailu et al., 2003a), *Jinzhousaurus* (Wang & Xu, 2001), and *Mantellisaurus atherfieldensis* (Norman, 1986; Paul, 2006) among others, but resting considerably lower than that of *Bactrosaurus johnsoni* (Godefroit et al., 1998), *Dakotadon lakotaensis* (Weishampel & Bjork, 1989; Paul, 2008), and *Ouranosaurus nigeriensis* (Taquet, 1976). A recurved premaxillary lip as seen in some hadrosaurid dinosaurs (Horner, Weishampel & Forster, 2004) is absent on *Choyrodon*, although the premaxillary shelf is demarcated by a raised ridge as in hadrosaurids (Horner, Weishampel & Forster, 2004). *Altirhinus* and *Iguanodon bernissartensis* lack the perimeter ridge (Norman, 1998). Posteriorly, lateral expansion of the oral margin is slight, lacking the abrupt postoral constriction of hadrosaurids species such as *Gryposaurus* spp. (Gates & Sampson, 2007), *Prosaurolophus maximus* (McGarrity, Campione & Evans, 2013), or even lambeosaurs (Evans & Reisz, 2007). Instead, the appearance is similar to *Altirhinus* (Norman, 1998), *Probactrosaurus* (Norman, 2002), and *Jinzhousaurus* (Wang & Xu, 2001; Barrett et al., 2009). The long, slender, moderately arching dorsal process is similar to, but distinct from that of *Altirhinus* (Norman, 1998), due to the larger external naris of the latter taxon. A large foramen rests at the base of the dorsal process. Another smaller foramen pierces the anterior surface, just dorsal to the oral margin.

The lateral process extends posterodorsally along the lateral skull, widening distally, yet does not form a lobate process as in *Eolambia caroljonesa* (McDonald et al., 2012a). A strong ridge propagates from the edge that bounds the premaxillary shelf to follow the dorsal margin of the lateral process throughout its length. This ridge appears stouter than that observed on other iguanodontians, although similar structures are known for *Probactrosaurus* (Norman, 2002), *Bactrosaurus* (Godefroit et al., 1998), *Dakotadon* (Weishampel & Bjork, 1989; Paul, 2008), *Equijubus* (Hailu et al., 2003a), and hadrosaurids.

**Figure 5:**
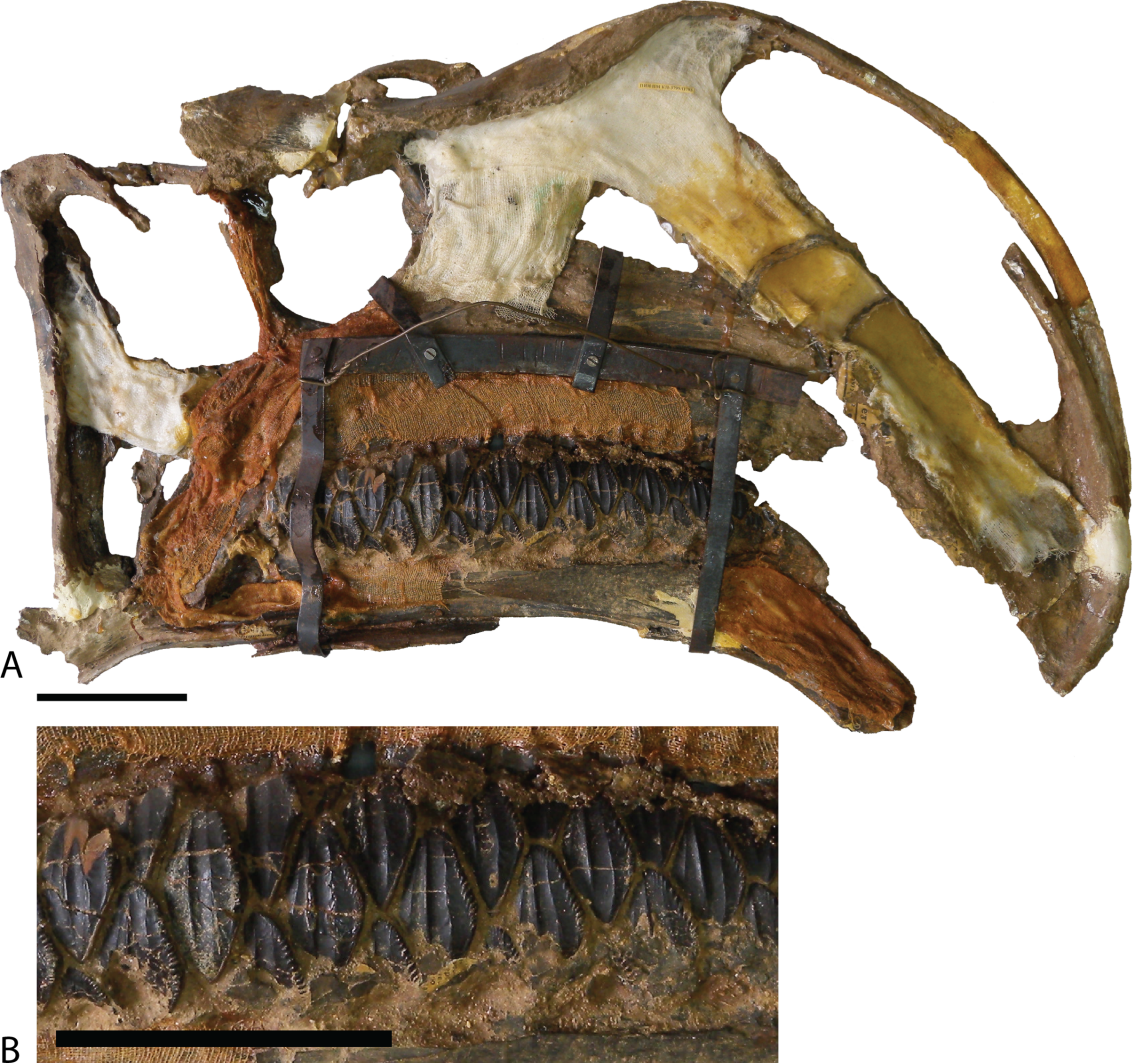
Type specimen of *Altirhinus*. (A) In medial view; (B) dentary teeth in medial view. Scale bars equal 10 cm. Photograph credit: Khishigjav Tsogtbaatar.

Maxilla—Similar to most other iguanodontians, the maxilla of *Choyrodon* is triangular and highly asymmetrical (Fig. 3). The element is mediolaterally narrow throughout its length, compared to the stouter hadrosaurid maxilla. Also, there are only 20 tooth positions, compared to ∼15 for *Jinzhousaurus* (Barrett et al., 2009), 21 in *Altirhinus* (Norman, 1998), 33 in the type specimen of *Eolambia* (McDonald et al., 2012a), and well over 40 known in some hadrosaurid taxa (Horner, Weishampel & Forster, 2004), although this is an ontogenetically variable character. A complex element, the maxilla is here divided into anterior, central, and posterior regions. The anterior portion slopes slightly anteroventrally to underlie the lateral process of the premaxilla. Additionally, dorsal and ventral anterior processes project from the medial side of the maxilla, slotting into receptive grooves on the medial side of the premaxilla. These processes are ubiquitous among iguanodontians. In *Choyrodon* the dorsal anterior process is relatively larger and more prominent than on *Proa valdearinnoensis* (on which this feature is virtually nonexistent; McDonald et al., 2012b), *Ouranosaurus* (this process is a small extension from the maxilla; T. Gates, 2014, personal observation), *Equijubus* (the feature on this taxon is short anteroposteriorly; T. Gates, 2014, personal observation), *Eolambia* (the process is long, moderately dorsoventrally expanded, similar to hadrosaurids; Kirkland, 1998; McDonald et al., 2012a), *Bactrosaurus* (Godefroit et al., 1998; Prieto-Márquez, 2011) and hadrosaurine hadrosaurids (the anterodorsal process in hadrosaurids is typically long and narrow, quite unlike the form seen on *Choyrodon* (e.g., *Gryposaurus* (Gates & Sampson, 2007), *Probrachylophosaurus* (Fowler & Horner, 2015); note that lambeosaurine hadrosaurids lack this process). A comparison with *Altirhinus* (PIN 3386; Fig. 5) shows that both taxa have shortened anterior maxillary processes. Reinforcement of PIN 3386 however covers over the section of the anterodorsal process that rises to meet the ventral border of the narial fenestra, making further comparison impossible. In fact, the dorsal anterior process of *Choyrodon* may be the largest relative to maxilla size for an iguanodontian yet described, having a height to length ratio of 1 when one measures dorsally at a point originating at the posteroventral-most point of the process. Also, as seen in Figs. 3C and 3D the anterodorsal maxillary process rises to the level of the ventromedial bony nares, following the contour of the fenestra for a short distance before descending back towards its anterior termination. Posterior to this process the dorsal surface of the maxilla is horizontal, a condition similar to *Proa* (McDonald et al., 2012b) except that there is more texture to the latter taxon. Again, comparing to *Altirhinus*, the latter species has an inclined dorsal border of the maxilla (Fig. 5), not horizontal as seen in *Choyrodon*.

The dorsal process is the most prominent feature of the central region. It is dorsally high and expansive, with the apex of the process positioned posterior to the middle of the maxilla. The premaxillary lateral process rides over the anterior surface of the dorsal process. The lacrimal contacts the dorsal process posteriorly through a depressed region on the expanded posterodorsal apex (this contact is smaller than in *Altirhinus*, Norman, 1998); the latter feature forms the anterior wall of the antorbital fenestra (see Lacrimal below). A large groove runs anteroposteriorly between the medial surface of the element and the dorsal process.

Numerous foramina mark the surface of the lateral maxilla, lying in a straight line near the posterior tooth positions. Four elements contact the posterior region of the maxilla: the jugal, palatine, pterygoid, and ectopterygoid. A sinuous groove marks the contact surface of the jugal, ending on the lateral-most extent of a prominent jugal process. As in other basal iguanodontians, the ectopterygoid rests on a small posteriorly dipping shelf, just posterior to the jugal contact. Hadrosaurids concomitantly increase the size of the shelf along and rotate the feature to a horizontal orientation. A substantial concavity in the anterior wall of this shelf receives the anterior process of the ectopterygoid, positioning the latter element to contact the jugal. The posteromedial ridge houses the small bulging palatine and pterygoid processes, the latter of which is not well preserved on MPC-D 100/801. The medial face of the maxilla is relatively flat, adorned by an arching row of dental foramina.

**Figure 6:**
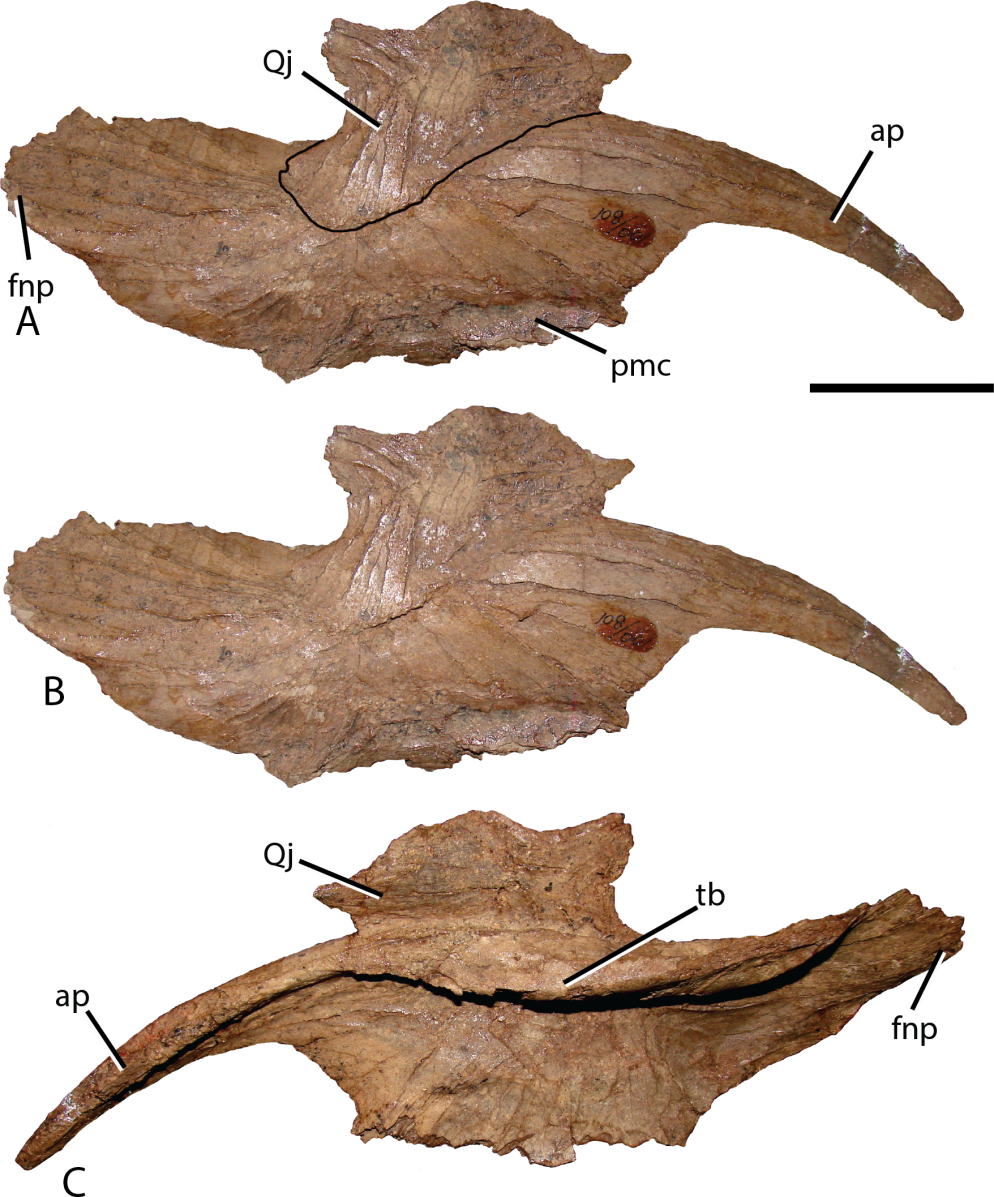
Isolated right nasal and quadratojugal of *Choyrodon* specimen MPC-D 100/801. (A) lateral with an outline of the quadratojugal; (B) lateral without an outline of the quadratojugal and (C) medial view. The right quadratojugal is closely adhered to the nasal of MPC-D 100/801 due to taphonomic processes. Study sites: ap, anterior process; avnp, anteroventral nasal process; fnp, frontonasal process; pmc, premaxillary contact; tb, thickened tear-drop bone. Scale bar equals five cm. Photograph credit: Terry Gates.

**Figure 7:**
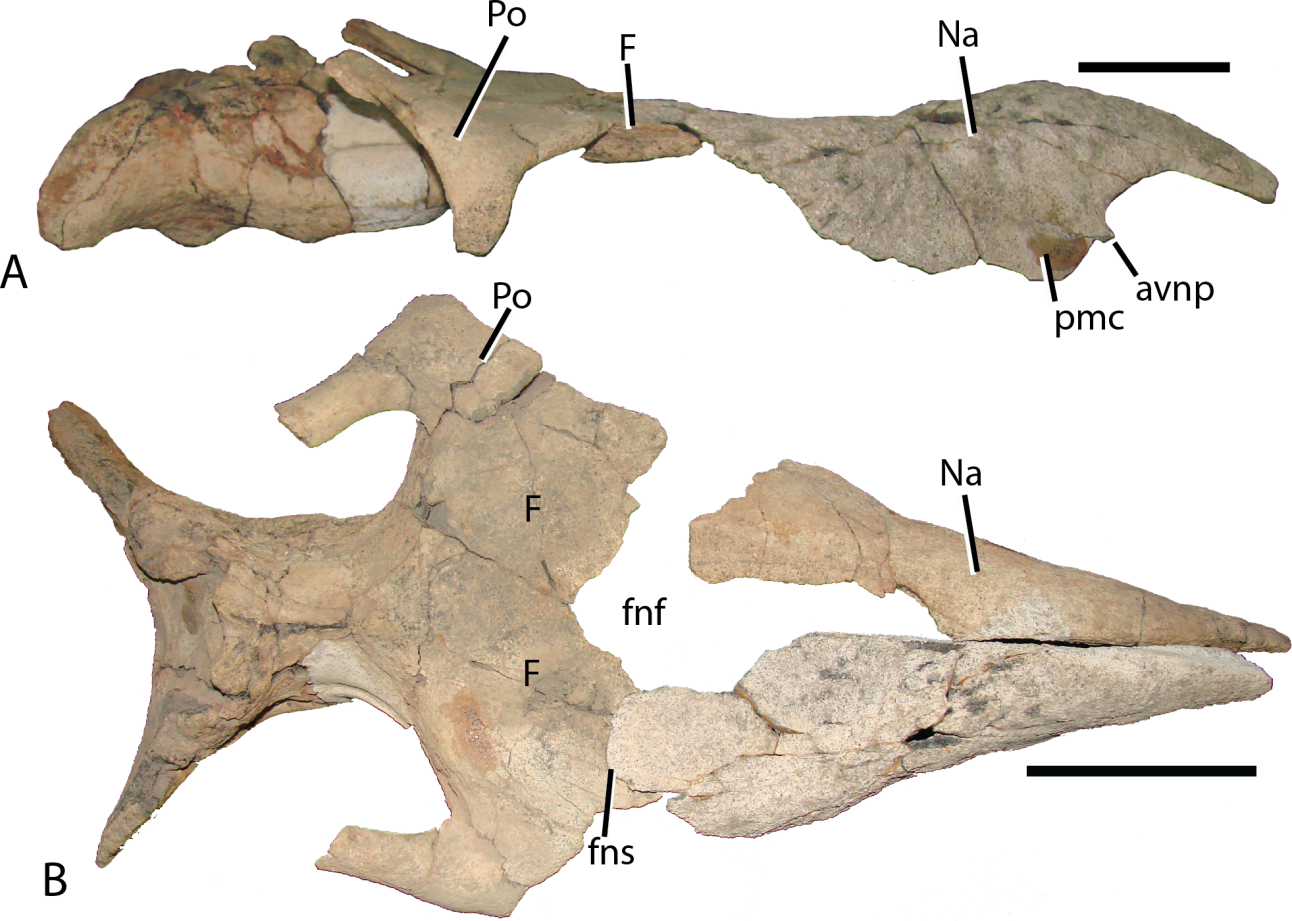
Reconstructed skull roof and nasal of *Choyrodon* specimen (MPC-D 100/800). (A) lateral view, and (B) dorsal view. The frontonasal fontanel is identified based on smooth unbroken edges on both the frontal and corresponding nasal. Study sites: avnp, anteroventral nasal process; F, frontal; fnf, frontonasal fontanel; fns, frontonasal suture; Na, nasal; pmc, premaxillary contact; Po, postorbital. Scale bar equals 10 cm. Photograph credit: Terry Gates.

Nasal—The nasal (Figs. 6 and 7) is one of the most unique bones in the skull of *Choyrodon*. The anteriorly projecting anterior nasal process terminates along the dorsal margin of the external nares, a feature common to all iguanodontians except lambeosaurine hadrosaurids and specific hadrosaurine taxa such as *Prosaurolophus*, *Saurolophus*, and *Edmontosaurus* (Gates & Sampson, 2007). In *Choyrodon* and other basal iguanodontians the anterior process terminates approximately midlength of the external nares whereas in hadrosaurids such as *Gryposaurus* this same process terminates much closer to the nares anterior border (Gates & Sampson, 2007). *Choyrodon* possesses a large internasal groove along the sagittal suture of the anterior process, as in *Altirhinus* (Norman, 1998). The nasal makes up the entire posterior border of the external nares, as well as rounding around a short section of the ventral border as in *Jinzhousaurus* (Barrett et al., 2009), *Altirhinus* (sensu Barrett et al., 2009), and some hadrosaurids. The posterior margin is straight and posteroventrally oriented, as opposed to the curved condition observed in all other taxa (except *Dakotadon* that possesses a straight posterior margin oriented posterodorsally; Weishampel & Bjork, 1989). A small groove demonstrates contact with the premaxilla lateral process along the posteroventral margin of the nasal, just beyond the external nares. The lacrimal contacted the nasal near this same margin, whereas the relatively minor contact with the prefrontal occurred along the posterolateral border. *Choyrodon* shares with *Altirhinus* (Norman, 1998) the same characteristic of lacking a depression on nasal posterior to the nasal fenestra. The dorsal surface of the nasal rises to form a small, slightly anteroposteriorly compressed, prominence. *Altirhinus* is diagnosed in part based on the large nasal arch (Norman, 1998), which is considerably larger than in *Choyrodon*, although this feature is known to make dramatic changes through ontogeny in hadrosaurids (see Discussion). The nasal prominence descends posteriorly into a shallow depression. Directly ventral to the depression, the undersurface of the nasal is thickened, which when paired with its counterpart, would have formed a teardrop shaped swelling, with the narrowest portion oriented anteriorly. *Gryposaurus* thickens its nasal in a similar position (E. Freedman-Fowler, 2014, personal communication). The nasal articulated with the frontal by overlapping onto a small lobate depression on the anterolateral corner of the frontal, a condition nearly identical to that described in *Altirhinus* (Norman, 1998) and *Jinzhousaurus* (Barrett et al., 2009), yet also similar to that of *Prosaurolophus* (Horner, 1992; McGarrity, Campione & Evans, 2013). The nasals do not contact along the midline posteriorly, leaving a fontanelle between the nasals and the frontals (Fig. 7). Frontonasal fontanels have been described in *Altirhinus* (Norman, 1998), *Bactrosaurus* (Godefroit et al., 1998), *Levnesovia* (Sues & Averianov, 2009), the hadrosauroid *Lophorhothon atopus* (Langston, 1960), and the hadrosaurid *Eotrachodon orientalis* (Prieto-Marquez, Erickson & Ebersole, 2016). The frontonasal fontanelle described by Norman (1998) is based on a specimen collected from a different site than the type specimen and, based on measurement from the original publication, is an equivalent size to MPC-D 100/801. This means that the fontanelle might diagnose PIN 3387 as *Choyrodon* or that the feature is also present in *Altirhinus* at an earlier stage of development than the holotype. Both fontanels are of a similar size and shape.

**Figure 8:**
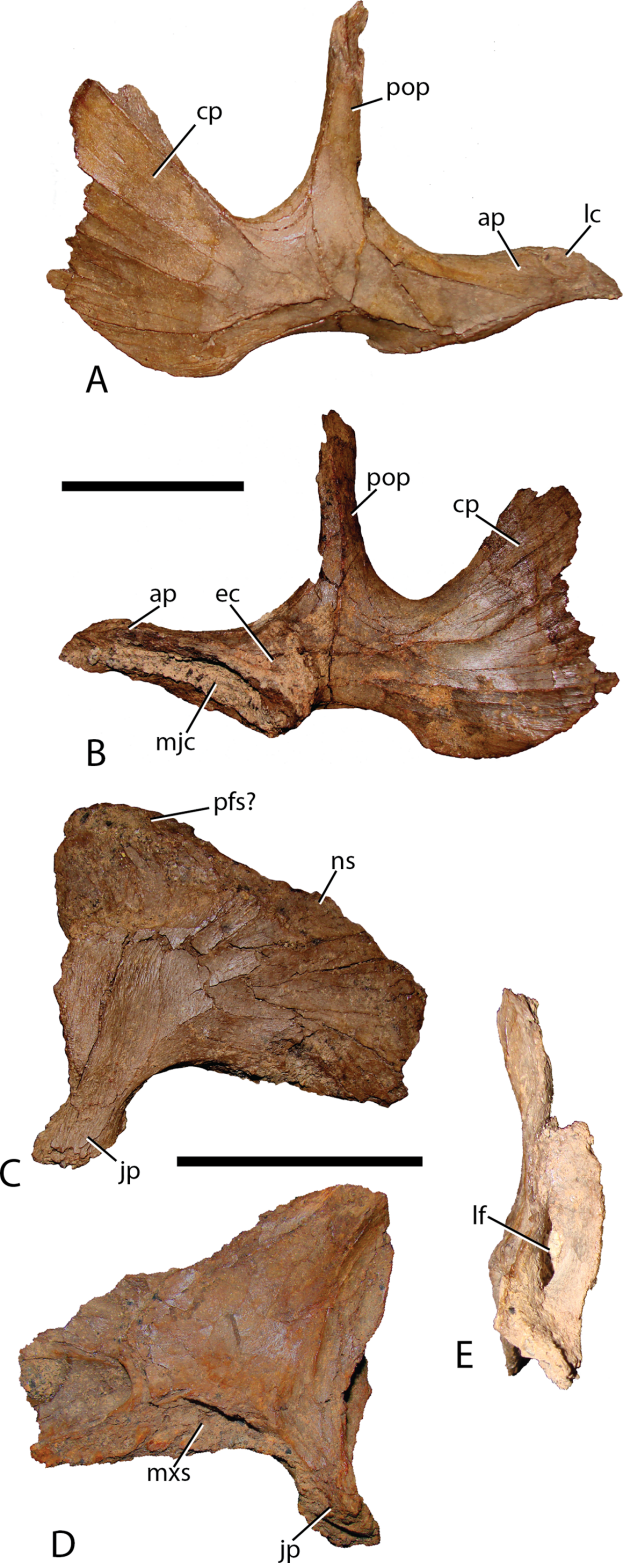
Jugal and lacrimal of *Choyrodon* specimen MPC-D 100/801. Right jugal in (A) lateral and (B) medial views; right lacrimal of MPC-D 100/801 in (C) lateral, (D) medial, and (E) posterior views. Study sites: ap, anterior process; cp, caudal process; ec, ectopterygoid contact; jp, jugal process; lc, lacrimal contact; lf, lacrimal foramen; mjc, maxilla-jugal contact; mxs, maxilla suture; ns, nasal suture; pfs, prefrontal suture; pop, postorbital process. Scale bars equal five cm. Photograph credit: Terry Gates.

Jugal—The jugal (Fig. 8) exhibits morphology common to many basal iguanodontians in that the anterior process is elongate, distally tapering, with minor dorsoventral expansion. The sigmoidal maxillary suture of the jugal broadly contacts the maxilla at a depression on the medial side of the jugal anterior process. The slender ventral margin of this process also slides into a receptive groove on the maxillary jugal process. The anterior-most region of the anterior process dorsal surface is slightly depressed for articulation with the lacrimal. The morphology displayed in *Choyrodon*, along with numerous other basal iguanodontians, is midway through the transformation from that observed in basal ornithopods (Norman, 2004) to that found in hadrosaurids (Horner, Weishampel & Forster, 2004). Medially, posterior to the maxilla suture, a triangular depression marks the contact with the conical process of the ectopterygoid. Unlike *Choyrodon*, *Altirhinus* possesses a rounded ectopterygoid process on the jugal (Norman, 1998). This feature is absent in more derived iguanodontians such as *Bactrosaurus* (Godefroit et al., 1998; Prieto-Márquez, 2011), *Telmatosaurus* (Weishampel, Norman & Grigorescu, 1993), and hadrosaurids (Horner, Weishampel & Forster, 2004). The postorbital process rises perpendicular to the jugal body, as in virtually all iguanodontians except some hadrosaurids (Gates & Sampson, 2007). The ventral margin of the jugal body is concave, accentuated by the slight ventral expansion of the jugal head and the caudal process. A posteroventral (i.e., free) flange is barely discernable on the ventral margin of the caudal process, but is more accentuated than that on *Muttaburrasaurus langdoni* (Bartholomai & Molnar, 1981), *Equijubus* (Hailu et al., 2003a), *Probactrosaurus* (Norman, 2002), *Bactrosaurus* (Godefroit et al., 1998; Prieto-Márquez, 2011), or *Eolambia* (Kirkland, 1998; McDonald et al., 2012a). The caudal process arches posterodorsally; unfortunately, this margin is not preserved on any *Choyrodon* specimen and the full extent of its morphology cannot be determined. The ventral margin of the infratemporal fenestra, created by the angle between the postorbital and caudal processes, is narrower than that described in *Altirhinus* (Norman, 1998; Fig. 4) and *Protohadros byrdi* (Head, 1998), being more similar to *Jinzhousaurus* (Wang & Xu, 2001; Barrett et al., 2009) and *Eolambia* (Kirkland, 1998; McDonald et al., 2012a).

**Figure 9:**
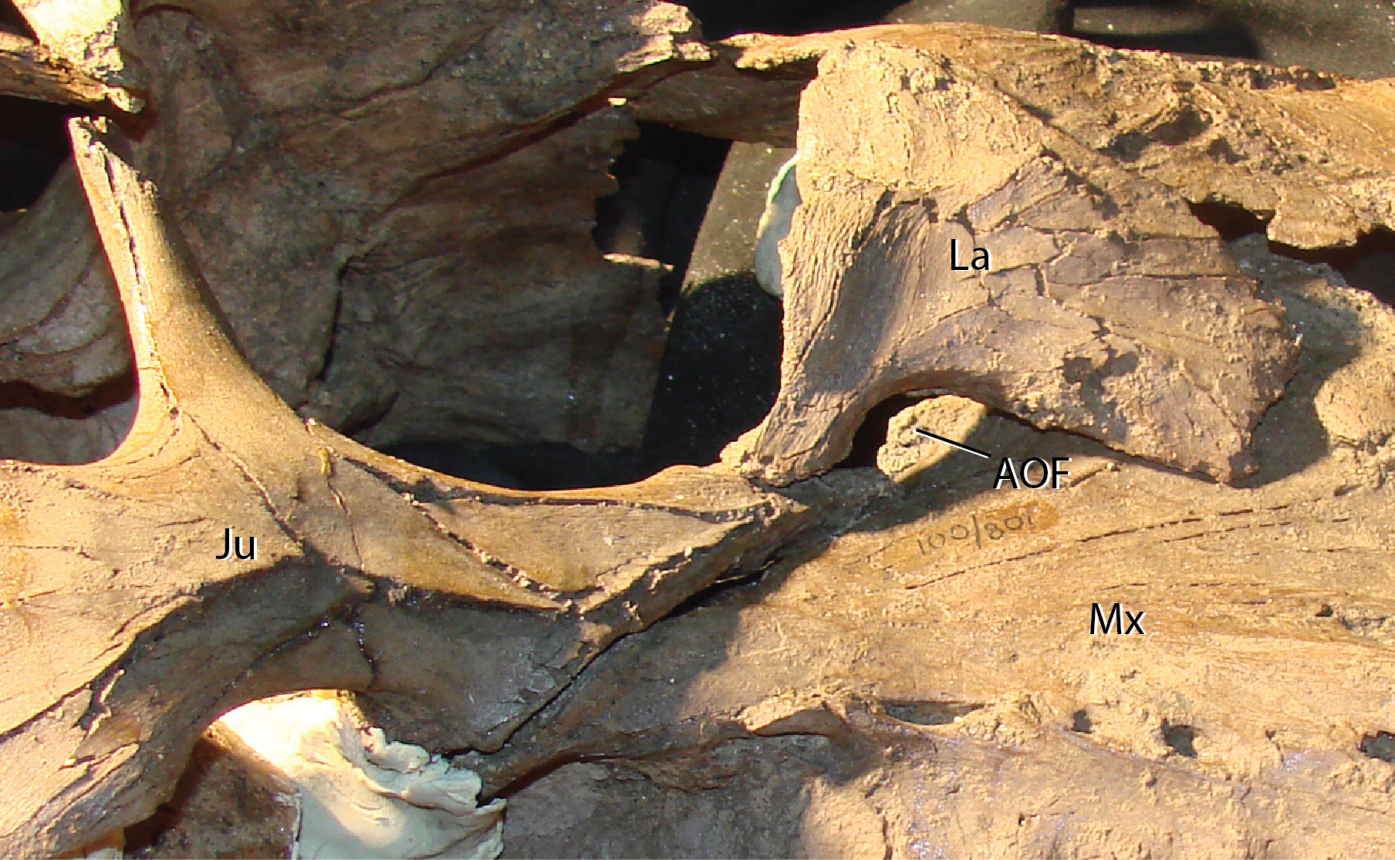
Maxilla, jugal, and lacrimal of *Choyrodon* specimen MPC-D 100/801 in articulation to show the antorbital fenestra. Study sites: Aof, antorbital fenestra; Ju, jugal; La, lacrimal; Mx, maxilla. Photograph credit: Terry Gates.

Lacrimal—Generally, the lacrimal (Fig. 8) is triangular in lateral view, with a large, columnar jugal process descending posteroventrally from the posteroventral corner. This simple morphology is different from that observed in all other iguanodontians. Minor damage to the anterior-most region prevents observation of the complete anterior morphology, but based on the position of sutures it seems that the preserved portion is well representative of the lacrimal orientation in life. The lacrimal extended to contact the premaxilla lateral process as in virtually all other iguanodontians, where the anterodorsal margin of the medial lacrimal surface possesses a deep suture that plausibly incorporated the premaxilla. Posteriorly along the same dorsal margin, the nasal slots into an enlongate groove on the lateral face of the lacrimal as in *Altirhinus* (Norman, 1998) and hadrosaurids (e.g., *Prosaurolophus*, McGarrity, Campione & Evans, 2013). *Xuwulong yueluni* (Hailu, Daqing & Weichang, 2011) also has a lacrimal that contacts the nasal; however, the suture is much shorter than in *Choyrodon*. Other species such as *Mantellisaurus* (Norman, 1986), *Iguanodon* (Norman, 1980), *Bolong yixianensis* (Wenhao & Godefroit, 2012), *Jinzhousaurus* (Barrett et al., 2009), and *Equijubus* (Hailu et al., 2003a) lack a nasal and lacrimal articulation. In medial view, the lacrimal articulates with the maxilla via reception of the maxillary dorsal process into a shallow triangular fossa positioned on the lacrimal anteromedial face. *Altirhinus* has a similar articulation of the lacrimal to the maxilla (Norman, 1998) as well as *Jinzhousaurus* (Barrett et al., 2009) and *Eolambia* (McDonald et al., 2012a). This configuration of the lacrimal articulation to dorsal process differs from that of *Iguanodon* and *Mantellisaurus* in that the latter two taxa have a narrow process extending posterodorsally that unites with the lacrimal. Norman (1998) describes the maxillary dorsal process as finger-like, however, it differs dramatically from that of *Iguanodon* and *Mantellisaurus*, which are both finger-like. Here, we describe the type of articulation facet between the maxilla and lacrimal as seen on *Choyrodon* or *Altirhinus* as wedge-shaped. The anterior margin of the jugal process is smooth, designating the posterior margin of the antorbital fenestra (Fig. 9). Of those iguanodontian species possessing an antorbital fenestra, the jugal process of *Iguanodon* (Norman, 1980), *Mantellisaurus* (Norman, 1986), and *Dakotadon* (Weishampel & Bjork, 1989) descends posteroventrally (Norman, 1980, 1986; Weishampel & Bjork, 1989) forming a broad oblique angle, like the process of *Choyrodon*. Iguanodontian taxa such as *Altirhinus* (Norman, 1998; Fig. 4B; note that preservation of this area is poor, but it appears that there is not one based on the position of the lacrimal), *Jinzhousaurus* (Wang & Xu, 2001; Barrett et al., 2009), *Bolong* (Wenhao & Godefroit, 2012), *Xuwulong* (Hailu, Daqing & Weichang, 2011), and *Equijubus* (Hailu et al., 2003a) lack an antorbital fenestra, along with more derived taxa such as *Telmatosaurus* (Weishampel, Norman & Grigorescu, 1993) and hadrosaurids (Horner, Weishampel & Forster, 2004). Note that close examination of Figs. 4 and 5 reveals glue and fabric covering the articulation between the maxilla and lacrimal on the *Altirhinus* specimen PIN 3386, obscuring the true nature of the antorbital fenestra both medially and laterally. The posterior face of the lacrimal is broad, containing the large opening for the lacrimal foramen. Dorsal to the foramen, the posterior surface is stepped, created by the immediate mediolateral constriction of the lacrimal.

**Figure 10:**
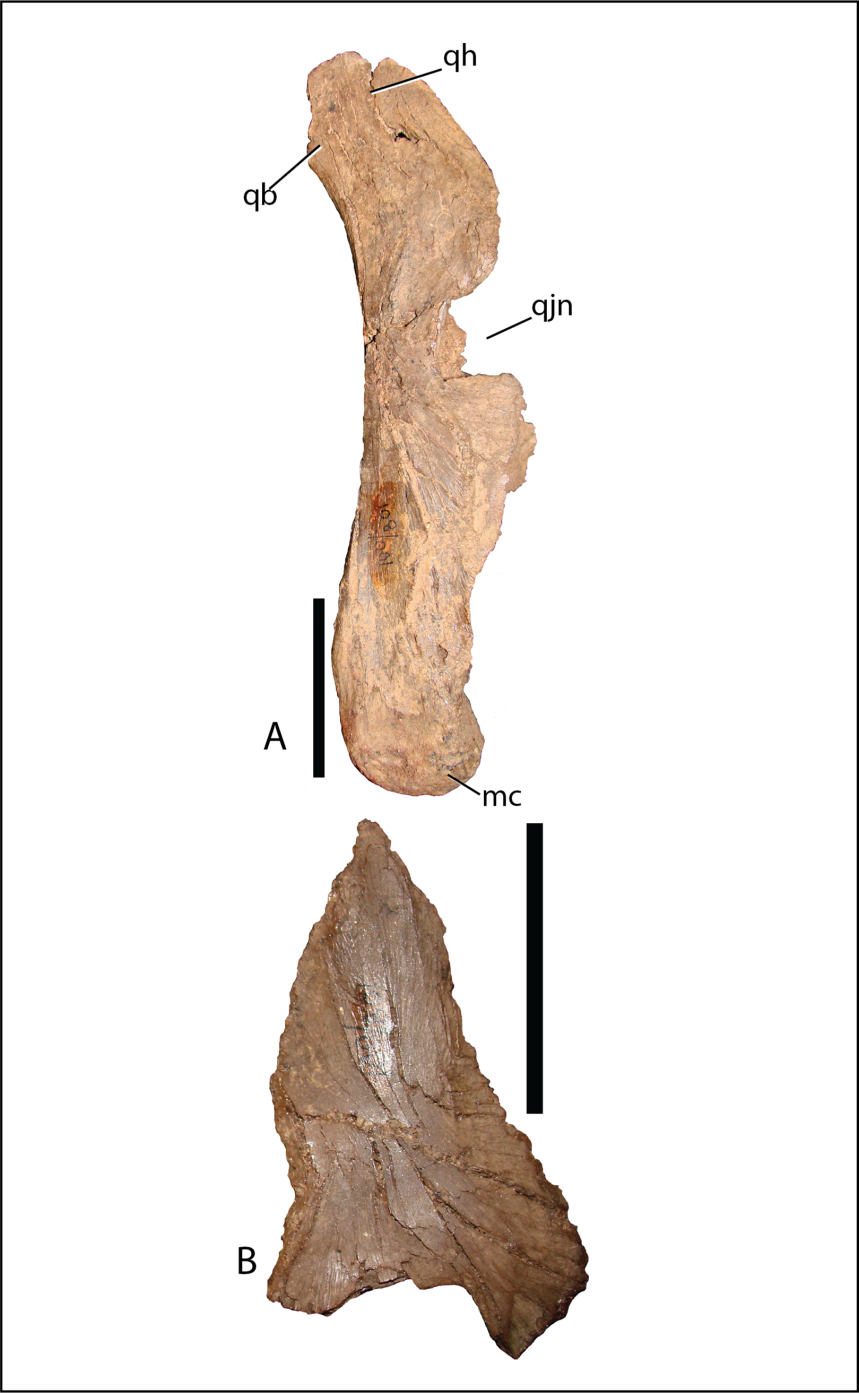
Quadrate (A) and (B) quadratojugal of *Choyrodon* specimen MPC-D 100/801 in lateral view. Study sites: mc, mandibular condyle; qb, quadrate buttress; qh, quadrate head; qjn, quadratojugal notch. Scale bars equal five cm. Photograph credit: Terry Gates.

Quadrate—The quadrate shaft is slightly posteriorly concave and articulates with the squamosal dorsally, differing from *Altirhinus* PIN 3386, which has a straight shaft throughout (Figs. 4 and 5). A long, slender, sharp-edged quadrate buttress (Fig. 10A) descends from the posterodorsal side of the shaft, yet is not as robust as in hadrosaurids like *Brachylophosaurus canadensis* or *Gryposaurus* spp. (Prieto-Márquez, 2005; Gates & Sampson, 2007). The paraquadratic notch, positioned just over half way up the shaft, is narrow, slightly asymmetrical, and possesses a dorsally oriented flange along its ventral margin in specimen MPC-D 100/801 akin to *Altirhinus* (Norman, 1998) (in MPC-D 100/800 the ventral border of the paraquadratic notch is oriented anteroventrally). Narrow notches are seen in most basal iguanodontian species and in the hadrosaurid *Velafrons coahuilensis* (Gates et al., 2007). The morphology of this notch is quite similar to *Altirhinus* (Norman, 1998), *Muttaburrasaurus* (Bartholomai & Molnar, 1981), and *Probactrosaurus* (Norman, 2002). A broad depression dorsal and ventral to the paraquadratic notch marks the contact surface between the quadrate and the quadratojugal. It is longer ventrally than dorsally. The mandibular condyles are reduced to one large condyle and a smaller medial one, similar to hadrosaurids (Horner, Weishampel & Forster, 2004) and differing from the two comparably sized condyles of *Altirhinus* (Norman, 1998), *Ouranosaurus* (Taquet, 1976), *Iguanodon* (Norman, 1980). The exact morphology of the pterygoid wing in both MPC-D 100/800 and MPC-D 100/801 is difficult to determine due to poor preservation, although the preserved portion of MPC-D 100/800 seems to suggest a broad feature with sloping dorsal and ventral margins that may have produced a rounded wing as seen in most hadrosaurids.

Quadratojugal—Generally, the quadratojugal (Figs. 6 and 10B) is long and triangular, the apex pointing dorsally, contacting the jugal anteriorly and the quadrate posteriorly. Both the anterior and posterior margins are sigmoid with the convex portion of one margin closely mimicking the opposing concave portion of the other. A dorsally directed, smooth surfaced indentation dominates the ventral margin. The quadratojugal predominantly occluded the paraquadratic notch of the quadrate, leaving only a narrow, dorsoventrally directed paraquadratic foramen. The size of this foramen seems to be smaller than that of *Equijubus* (Hailu et al., 2003a), *Muttaburrasaurus* (Bartholomai & Molnar, 1981), *Altirhinus* (Norman, 1998), *Iguanodon* (Norman, 1980), and *Mantellisaurus* (Norman, 1986).

Prefrontal—The only example of the prefrontal is a badly damaged strap-like element adhered to the maxilla on MPC-D 100/801. Little information can be gleaned from this element.

**Figure 11:**
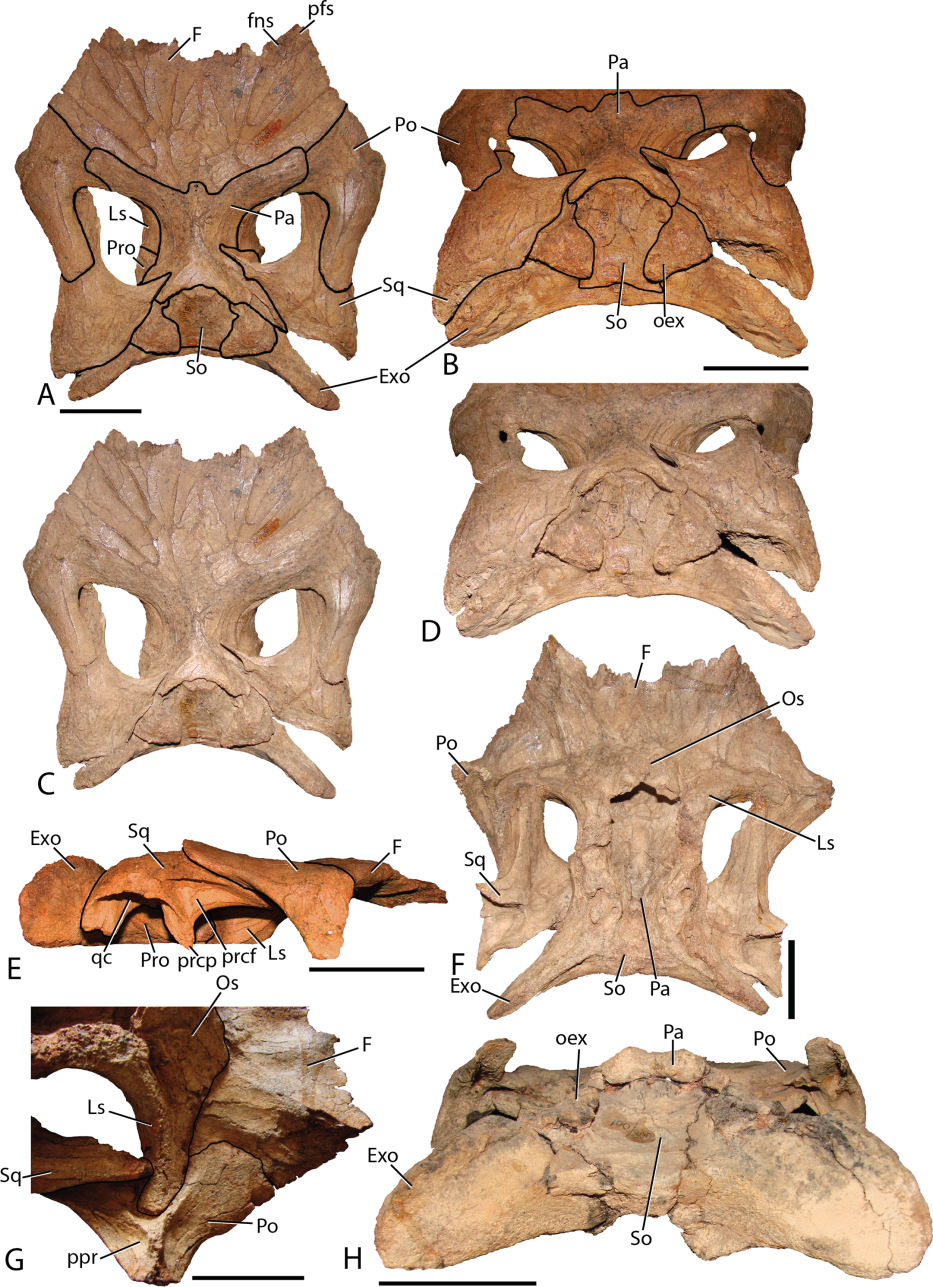
Skull roof of *Choyrodon*. (A) MPC-D 100/801 in dorsal view with lines depicting articulations between elements; (B) MPC-D 100/801 in posterodorsal view with lines depicting articulations between elements; (C) MPC-D 100/801 in dorsal view; (D) MPC-D 100/801 in posterodorsal view; (E) MPC-D 100/801 in right lateral view; (F) MPC-D 100/801 in ventral view; (G) MPC-D 100/801 in right lateroventral view; and (H) MPC-D 100/800 in posterior view. Study sites: Exo, exoccipital; F, frontal; fns, frontonasal suture; Ls, laterosphenoid; Os, orbitosphenoid; Pa, parietal; pfs, prefrontal suture; Po, postorbital; oex, opisthotic onlap of the supraoccipital; ppr, postorbital posterior recess; prcf, precotyloid fossa; prcp, precotyloid process; Pro, prootic; qc, quadrate cotylus; So, supraoccipital; Sq, squamosal. Scale bars equal five cm. Photograph credit: Terry Gates.

Frontal—The frontals (Fig. 11) are transversely wider than anteroposteriorly long and are slightly depressed medially. Each element possesses a shallow rounded depression, positioned on the anterolateral corner, for articulation with the nasal. This configuration is similar to several iguanodontians including *Jinzhousaurus* (Barrett et al., 2009). The entire anteromedial margin is devoted to the frontonasal fontanelle (see Nasal description above). Contacts for the prefrontal and postorbital (anteriorly and posteriorly, respectively) are separated by the lateral-most extent of the frontals, which also participates significantly in the orbital rim. Only *Jinzhousaurus* (Wang & Xu, 2001; Barrett et al., 2009) and some hadrosaurids (e.g., lambeosaurines, Evans & Reisz, 2007, *Prosaurolophus*, McGarrity, Campione & Evans, 2013) exclude the frontal from the orbital rim. Along the midline, the frontals appear to accept a small interdigitate frontal process from the parietal. Ventrally, the frontals are seen contacting the laterosphenoids and the orbitosphenoids in specimen MPC-D 100/801.

Postorbital—The postorbital (Fig. 11) contacts the frontal anteromedially and the parietal directly medially to the main elemental body. Its slightly rugose orbital rim is anteroposteriorly short due to the large frontal contribution to the orbital rim. On the medial surface a large rounded pocket accepts the laterosphenoid. Additionally, there is a shallow embayment on the temporal fenestra concavity at the ventral end on which a small foramen resides. Postorbital embayments are not common among iguanodontians. A majority of taxa have smooth temporal fenestrae surfaces. The most extreme example among iguanodontians is the hadrosaurid *Edmontosaurus*, which bears a hugely inflated pocket in the same position on the postorbital (Lambe, 1920; Campione & Evans, 2011). The posterior (squamosal) process of each postorbital rises significantly dorsally to contact the conjoining process from the squamosal (MPC-D 100/800 rises 158°, more steeply than MPC-D 100/801, 166°, measured from the dorsal surface of the horizontal frontal platform) to form an arched supratemporal bar. Dorsally angled posterior processes of the postorbital are found on *Iguanodon* (Norman, 1980), *Proa* (McDonald et al., 2012b), and *Xuwulong* (Hailu, Daqing & Weichang, 2011) among basal iguanodontians, and are more prevalent in hadrosaurids. *Gryposaurus* species and other closely related taxa have the most dramatic elevation of these processes (Gates & Sampson, 2007), demonstrating that this feature leads to a posterior skull that is much higher than the anterior portion. Farke & Herrero (2014) showed that ontogeny plays a critical role in angulation with larger (presumably older) individuals possessing more steeply inclined postorbital posterior processes. Additionally, the processes of *Choyrodon* inflect medially instead of simply posteriorly as in other taxa.

Squamosal—The squamosal (Fig. 11) meets the postorbital anteriorly (as described above). The precotyloid fossa is deep and triangular, similar to that described in the hadrosaurid *Gryposaurus monumentensis* (Gates & Sampson, 2007), *Rhinorex* (Gates & Scheetz, 2015), as well as *Altirhinus* (Norman, 1998; Fig. 4), *Proa* (McDonald et al., 2012b), and *Jinzhousaurus* (Wang & Xu, 2001; Barrett et al., 2009). This feature is nearly or completely suppressed in most other basal iguanodontian taxa and lambeosaurine hadrosaurids. The precotyloid process is small and triangular, residing just anterior to the deep quadrate cotylus. A long tapering strap-like median process abuts the parietal, which along with the supraoccipital, widely separates the squamosals in dorsal view. Separation of the squamosals is primitive among ornithopods, whereas more derived taxa such as hadrosaurids either unite the squamosals along the midline or separate them by means of a thin parietal ridge.

Parietal—The parietal (Fig. 11) is slightly hour-glassed shaped in dorsal view, with an interdigitate frontal process on the anteromedial margin. The prootic and laterosphenoid contact this element laterally. The parietal slopes posterodorsally, overlapping the supraoccipital posteriorly and rising above the level of the anterior skull roof in MPC-D 100/800 (although the same region is less exaggerated in MPC-D 100/801). A sharp suture on the posterodorsal corners of the parietal mark the insertion of the squamosal medial processes. There is no evidence of a sagittal crest on MPC-D 100/801, yet a broken surface seen on the dorsal rim of the MPC-D 100/800 parietal suggests at least a small crest in this taxon.

Orbitosphenoid—Observed only on MPC-D 100/801 (Fig. 11D), this element is positioned anteromedial to the laterosphenoid. The orbitosphenoid meets its partner medially and the frontals dorsally. Overall, the diamond shaped bone is similar to that of other iguanodontians and hadrosaurids.

Laterosphenoid—The preserved dorsal portion of the laterosphenoid (Fig. 11D) is morphologically similar to that seen in other iguanodontians and hadrosaurids. A long toothed margin typifies the dorsal articulation with the parietal and frontal, whereas a ball joint at the lateral-most extent inserts into a pocket on the postorbital. The prootic articulates with the posterior margin of the laterosphenoid. Overall, this element extends posteriorly to a greater relative distance (to at least half of the length of the dorsal temporal fenestra) than in other taxa such as the hadrosaurid *Acristavus gagslarsoni* (Gates et al., 2011) (only one-third the length of the dorsal temporal fenestra). There is no evidence of the foramen that held Cranial Nerve V in the preserved section. Anteriorly, the optic nerve (CN II) foramen is formed by the contact between the laterosphenoid and the orbitosphenoid.

Prootic—Not much information can be discerned about this element. On MPC-D 100/800 it is observed to be a blocky short bone with stubby posterior and posterodorsal processes that terminate a short distance from the dorsal border of the opisothotic, unlike the elongated gracile prootic of *Acristavus* (UMNH VP 16607; Gates et al., 2011).

Fused exoccipital-opisthotic—Though fusion prevents exact determination of each element, the most unique feature of the opisthotic is a large posterior process that drapes over to terminate on the posterior face of the supraoccipital (Fig. 11). This feature is prominent on MPC-D 100/801, and only slightly more subdued on MPC-D 100/800. Two species seem to have similar structures. The only specimen of *Yanganglong* (Wang et al., 2013) is badly eroded, yet there exists two prominent bulges symmetrically placed about the dorsal margin of the supraoccipital. No overlapping extension is seen on this specimen, however, such overlap is seen on *Jintasaurus* (You & Li, 2009, fig. 2).

The exoccipitals (Fig. 11) conjoin sagittally, forming the roof of the foramen magnum. The paroccipital processes contact the squamosal postcotyloid process. The anterior process of the exoccipital/opsithotic runs anteroventrally along the base of the prootic. Poor preservation prevents further description.

Supraoccipital—The supraoccipital of *Choyrodon* is inclined, as opposed to horizontal in hadrosaurids. In posterior view (Fig. 11), the supraoccipital is subtriangular in shape with a rounded dorsal region underlying the parietal. Two large bulbs protrude posteromedially, contacting the squamosals. The exoccipitals form a broad platform supporting the supraoccipital, preventing its inclusion within the dorsal margin of the foramen magnum.

### Palatoquadrate

**Figure 12:**
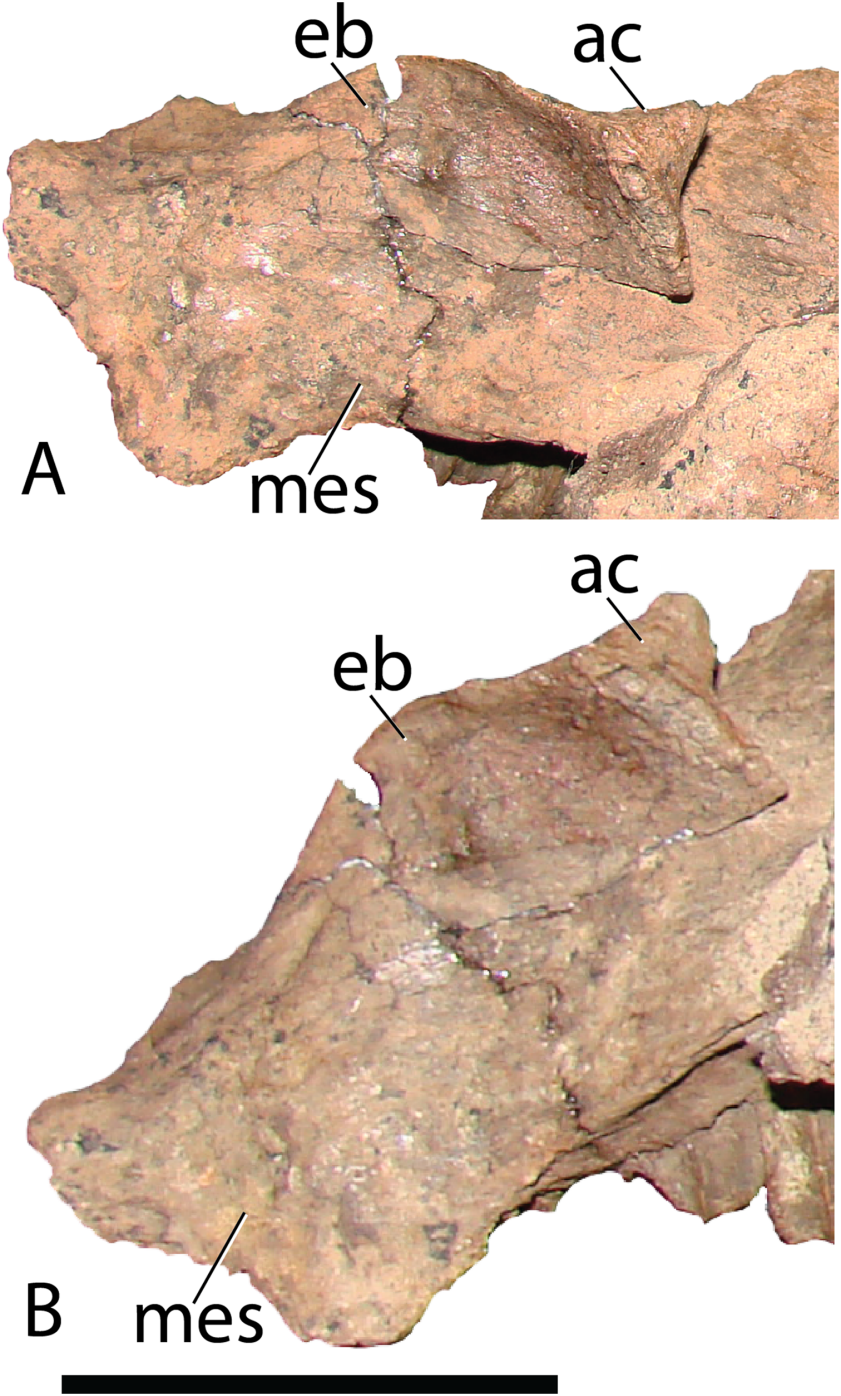
Ectopterygoid as preserved on the maxilla of *Choyrodon* specimen MPC-D 100/801. (A) anterodorsal view and (B) lateral view. Study sites: ac, anterior cone; eb, ectopterygoid body; mes, maxilla ectopterygoid shelf. Scale bar equals five cm. Photograph credit: Terry Gates.

Ectopterygoid—The strap-like, right ectopterygoid is preserved slightly displaced on the corresponding maxilla of MPC-D 100/801 (Fig. 12). Anteriorly, a large, conical process extends dorsally to contact the medial side of the jugal, as in other more basal iguanodontians, yet is subsequently lost in more derived taxa closer to hadrosaurids, such as *Bactrosaurus* (Godefroit et al., 1998; Prieto-Márquez, 2011), *Telmatosaurus* (Weishampel, Norman & Grigorescu, 1993), and *Protohadros* (Head, 1998). The anterior margin of the conical process inserts into a receptive notch in the maxilla. The pterygoid articulation is not apparent, however, according to Norman (1998), the ectopterygoid likely attached to the lateral aspect of the pterygoid.

**Figure 13:**
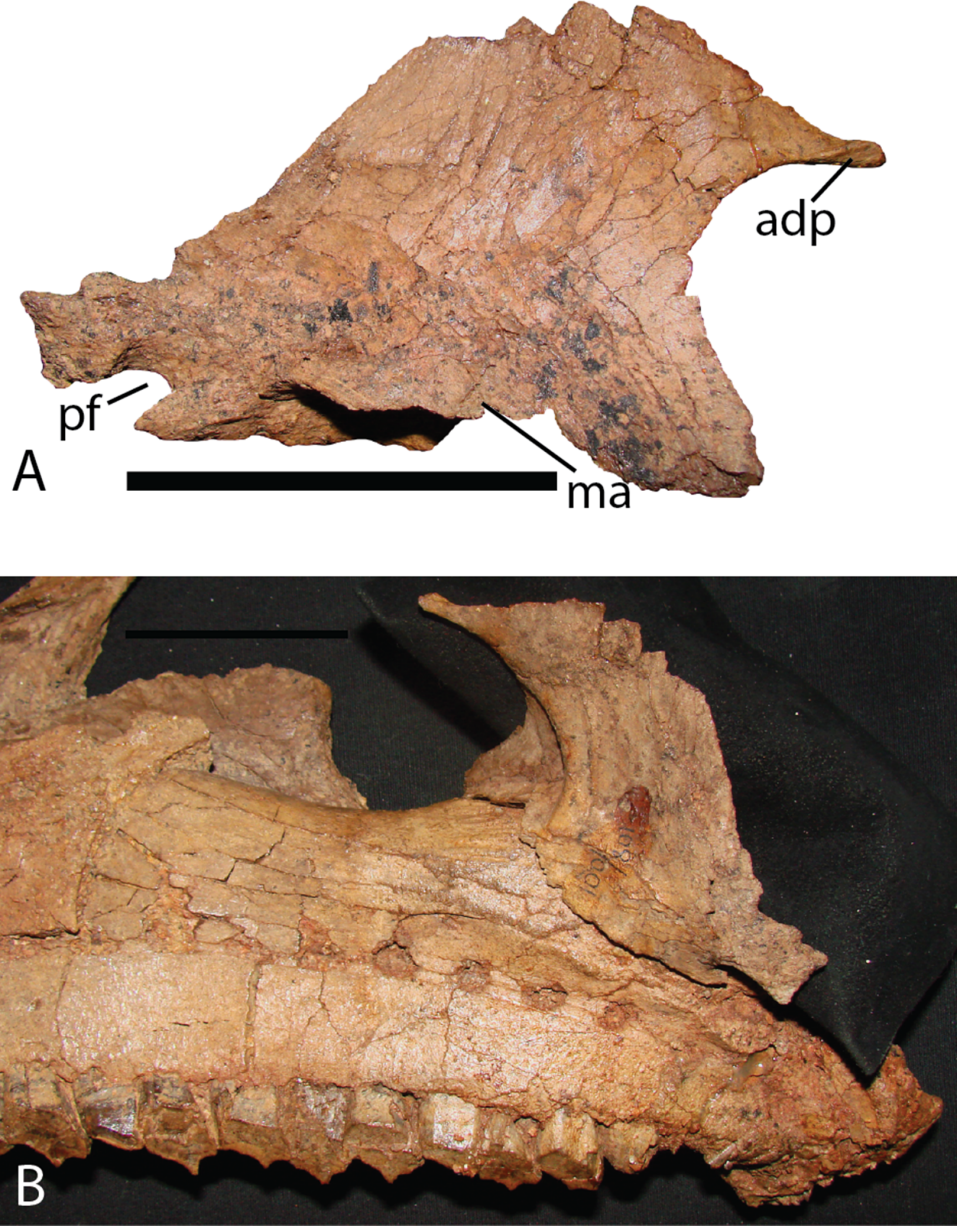
Palatine of *Choyrodon* specimen MPC-D 100/801. (A) lateral view; and (B) medial view articulating with maxilla. Study sites: adp, anterodorsal process; ma, maxillary articulation; pf, palatine foramen. Scale bar equals five cm. Photograph credit: Terry Gates.

Palatine—The right palatine (Fig. 13) is virtually complete on MPC-D 100/801, and generally similar to that described for *Altirhinus* (Norman, 1998), yet exhibits several notable distinctions. The palatine of *Choyrodon* is triangular. The anterodorsal corner expands into an elongate, narrow process, that is much more exaggerated than in *Altirhinus* (Norman, 1998). Additionally, the overall body shape has a higher profile than in the latter taxon. The ventral articulation is elongate, and concave. The center of the articulation is deepest to adhere firmly to the maxillary palatine process. This contact in *Choyrodon* differs from the anteroposteriorly compressed maxilla contact, and nearly “T” shaped profile of *Ouranosaurus* (T. Gates, 2014, personal observation). The anterior border has a deep accessory concavity on the medial side. The dorsal border sweeps ventrally toward the poster aspect, forming a steep arch where it terminates in the palatine foramen. The pterygoid suture occupies the posterior two-thirds of the dorsal arch, accompanying a large pterygoid buttress to hold the palatine more firmly to the pterygoid.

**Figure 14:**
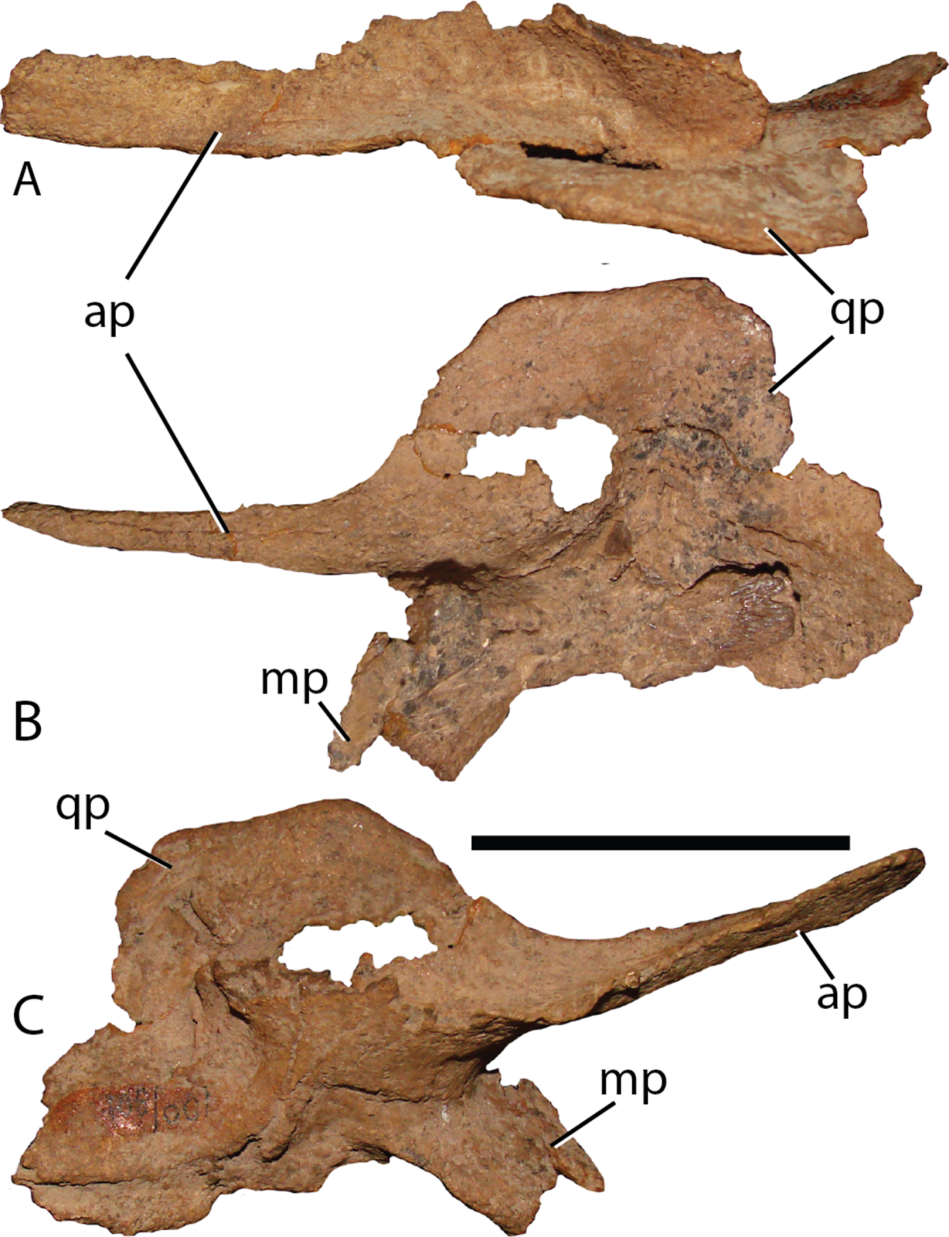
Pterygoid of *Chyrodon* specimen MPC-D 100/801. (A) dorsal; (B) lateral; and (C) medial views. Study sites: ap, anterior process; mp, maxillary process; qp, quadrate process. Scale bar equals five cm. Photograph credit: Terry Gates.

Pterygoid—Norman (1998) thoroughly details the morphology of the pterygoid of *Altirhinus*. The pterygoid of *Choyrodon* (Fig. 14) is similar in morphology, yet exhibits several differences. The dorsal wing is positioned more posteriorly, giving a broader exposure of the medial wing in lateral view. There is an acute angle between the medial and dorsal wing, as opposed to the right angle illustrated for *Altirhinus* (Norman, 1998). The dorsal ridge separating the medial and lateral regions of the pterygoid is curved medially, becoming concave in its anterior half, unlike the straight ridge in *Altirhinus* (Norman, 1998). Finally, a small process not described for *Altirhinus* projects from the ventral margin of the anteromedial side of the element.

### Mandibular complex

**Figure 15:**
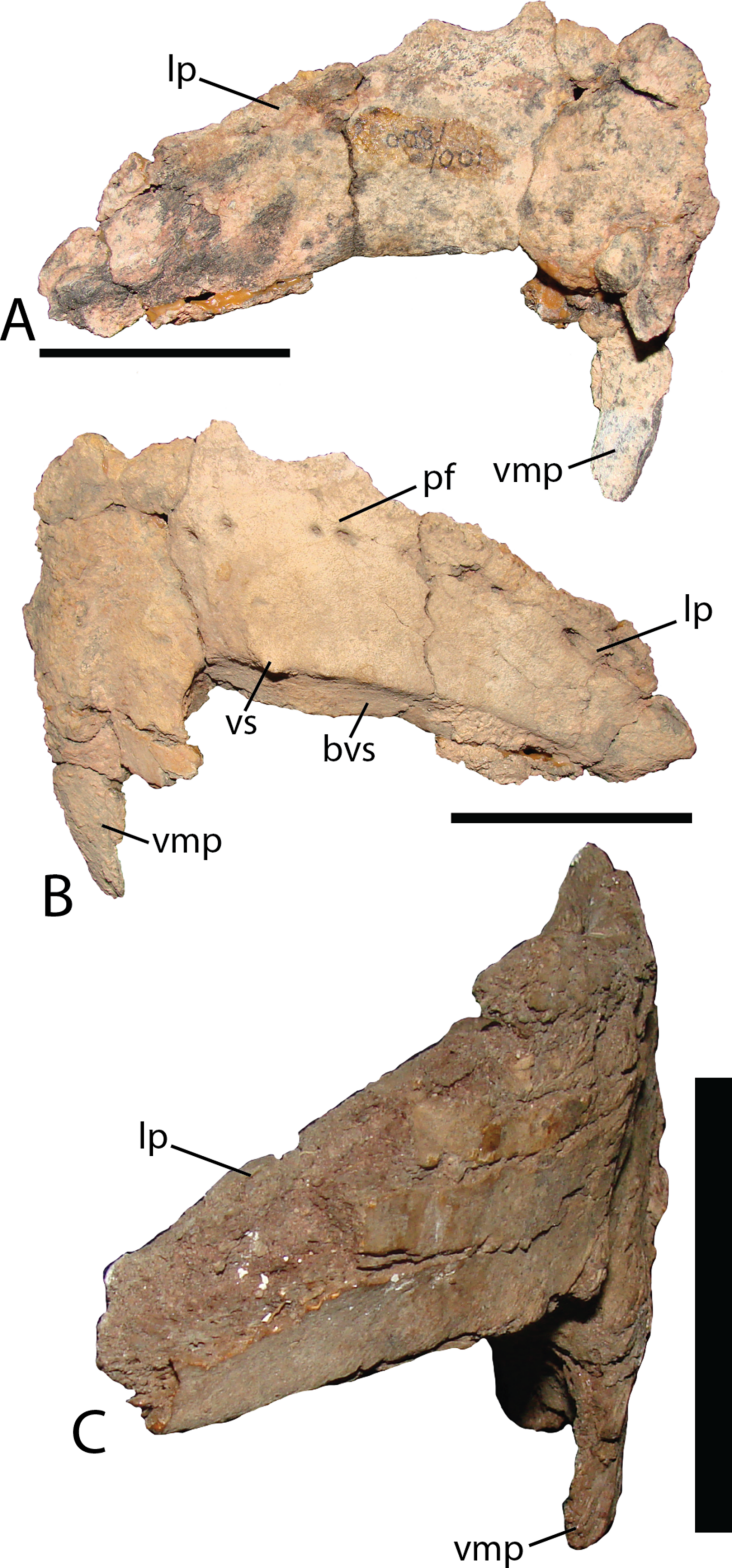
Predentary of *Choyrodon*. (A) MPC-D 100/800 in dorsomedial view; (B) MPC-D 100/800 in right ventrolateral view; (C) MPC_D 100/801 in lateral view. Study sites: bvs, beveled ventral surface; lp, lateral process; pf, paired foramina; vmp, ventromedial process; vs, ventral spikes. Scale bar equals five cm. Photograph credit: Terry Gates.

Predentary—MPC-D 100/800 contains the left half of a predentary (Fig. 15). Lateral processes extend posterolaterally, tapering along their length. From the midline, three prominent triangular labiolingually compressed prongs are present. More may have been present in life yet cannot be discerned due to poor preservation. A series of paired foramina continue from the midline to the posterior extent of the element, underlying the oral margin. The configuration of foramina (two holes being closely spaced and the next pair separated from the previous by a distance of three times that within each pair) is not described in *Altirhinus* (Norman, 1998), *Eolambia* (McDonald et al., 2012a), *Proa* (McDonald et al., 2012b), *Jinzhousaurus* (Barrett et al., 2009) or *Bolong* (Wenhao & Godefroit, 2012), and is otherwise a unique feature of *Choyrodon* to the best of our knowledge. In dorsal view the predentary would have been a tight arc more similar to *Proa* (McDonald et al., 2012b) than to *Altirhinus* (Norman, 1998), *Eolambia* (McDonald et al., 2012a), or *Mantellisaurus* (Norman, 1986). A long process descends from the anterior apex of the element, remaining slender throughout. This is in contrast with the distal expansion of this process on *Jinzhousaurus* (Barrett et al., 2009), *Altirhinus* (Norman, 1998), and *Proa* (McDonald et al., 2012b). As described by Norman (1998, fig. 15), *Altirhinus* possesses an anterior symphysis process that curves posteroventrally in a smooth arcuate fashion. Additionally, the *Altirhinus* anterior symphysial process bifurcates at its distal end, with an approximate distance between the main body of the predentary and the bifurcation of about one-half the depth of the anterior main predentary body. Current specimens of *Choyrodon* do not show a corresponding bifurcating feature, but instead show a straight process in two specimens. Even if the bifurcation were missing due to preservation, the orientation of the anterior symphysis process differs between *Choyrodon* and *Altirhinus*. The caudal symphysis process is shorter and more slender than the anterior symphysis process, descending in a similar fashion to *Eolambia* (McDonald et al., 2012a), not extending posteriorly in a horizontal fashion as described in *Bolong* (Wenhao & Godefroit, 2012). The base of the lateral process is flattened and angled more caudally than the anterior aspect. This region adheres to the dentary, and such morphology has not been described in another iguanodontian taxon. Three small spikes that decrease in size posteriorly are present at the junction between the ventral dentary articular surface and the anterior predentary surface.

**Figure 16:**
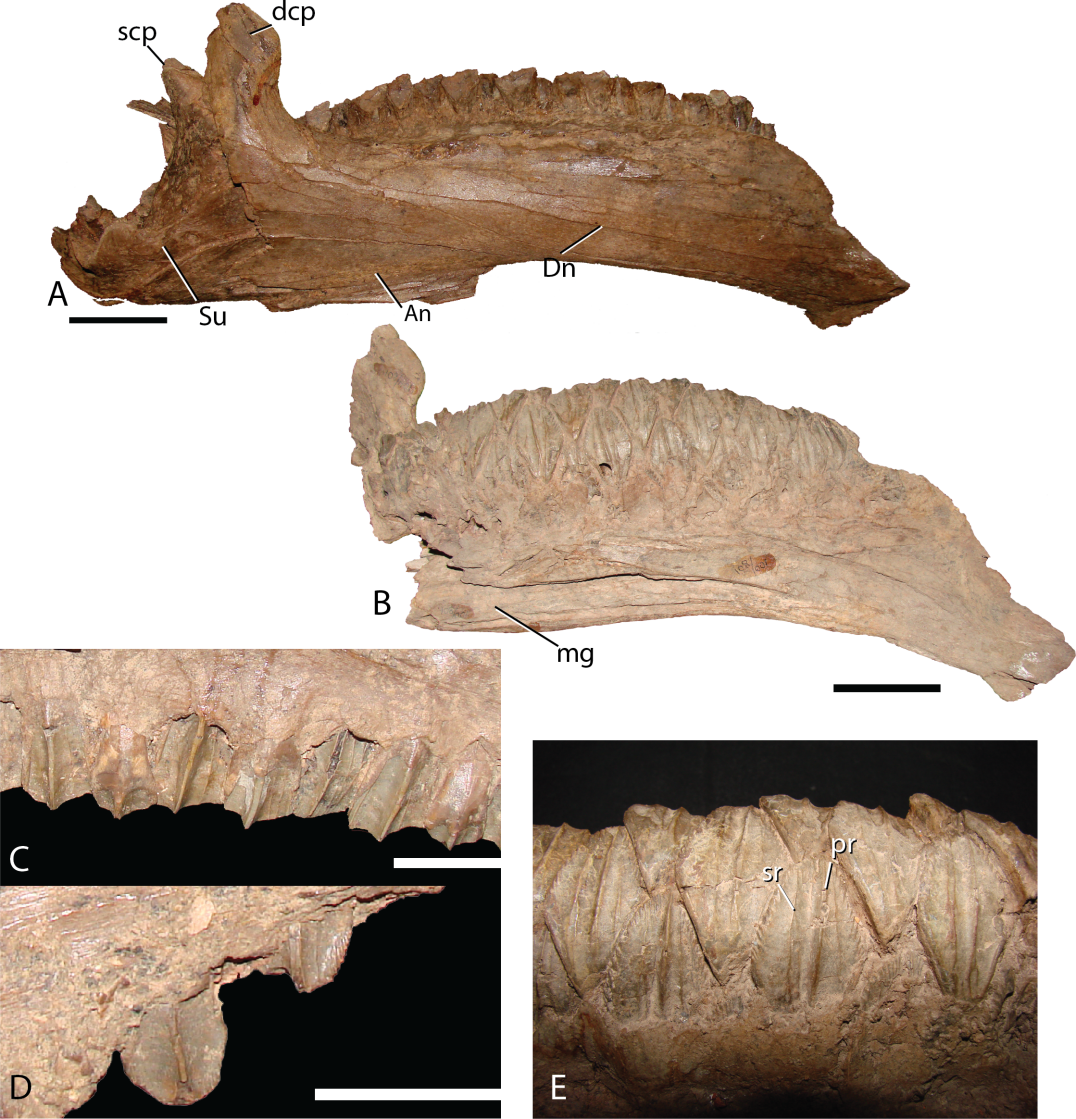
Dentary and teeth of *Choyrodon* MPC-D100/801. (A) MPC-D100/801 articulated right dentary and surangular in lateral view; (B) left dentary in medial view; (C) right maxillary teeth in lateral view showing midmaxillary examples; (D) right maxillary teeth in lateral view showing anterior example with denticles around crown; (E) right dentary teeth in lingual view. Study sites: An, angular; dcp, dentary coronoid process; Dn, dentary; mg, Meckelian groove; pr, primary ridge of dentary teeth; scp, surangular coronoid process; sr, secondary ridge of dentary teeth; Su, surangular. Scale bars in (A) and (B) equal five cm. Scale bars in (C) and (D) equal two cm. Photograph credit: Terry Gates.

Dentary—The dentary (Fig. 16A) of *Choyrodon* is more strongly downturned anteriorly than most other iguanodontians, except *Protohadros* (Head, 1998). In fact among basal iguanodontians, only *Altirhinus*, *Choyrodon*, and *Proa* (McDonald et al., 2012b), posses a downturned dentary that compares with hadrosaurids such as *Protohadros* all other iguanodontian taxa are characterized by straight dentaries. The early iguanodontian genera *Penelopognathus weishampeli* (Godefroit, Li & Shang, 2005), *Shuangmiaosaurus gilmorei* (Hailu et al., 2003b), and *Jayewati rugoculus* (McDonald, Wolfe & Kirkland, 2010) differ dramatically from *Choyrodon* in that all three have straight, thin dentaries. The dentary symphysis of *Choyrodon* is relatively narrow anteroposteriorly and notched ventrally for the reception of the predentary posteroventral flanges. Laterally, the surface of the dentary is pocked with an arched line of foramen that extends from the short diastema (two teeth wide) posteriorly to the coronoid process. The coronoid process juts laterally from the side of the dentary, shielding the final tooth of the dental battery from lateral view. The anterior margin of the spade-shaped coronoid process apex projects anteriorly, whereas the posterior side is grooved slightly for the reception of the coronoid process of the surangular, a trait ubiquitous among basal iguanodontians (Norman, 2004). Medially, the tooth row dominates the surface. There are 21 tooth positions in MPC-D 100/801 and only two teeth per family. The Meckelian groove extends almost the entire length of the tooth row. The posterolateral margin is broken so the morphology cannot be determined accurately; however, there is a large overlapping joint between the angular, which is observed in lateral view.

**Figure 17:**
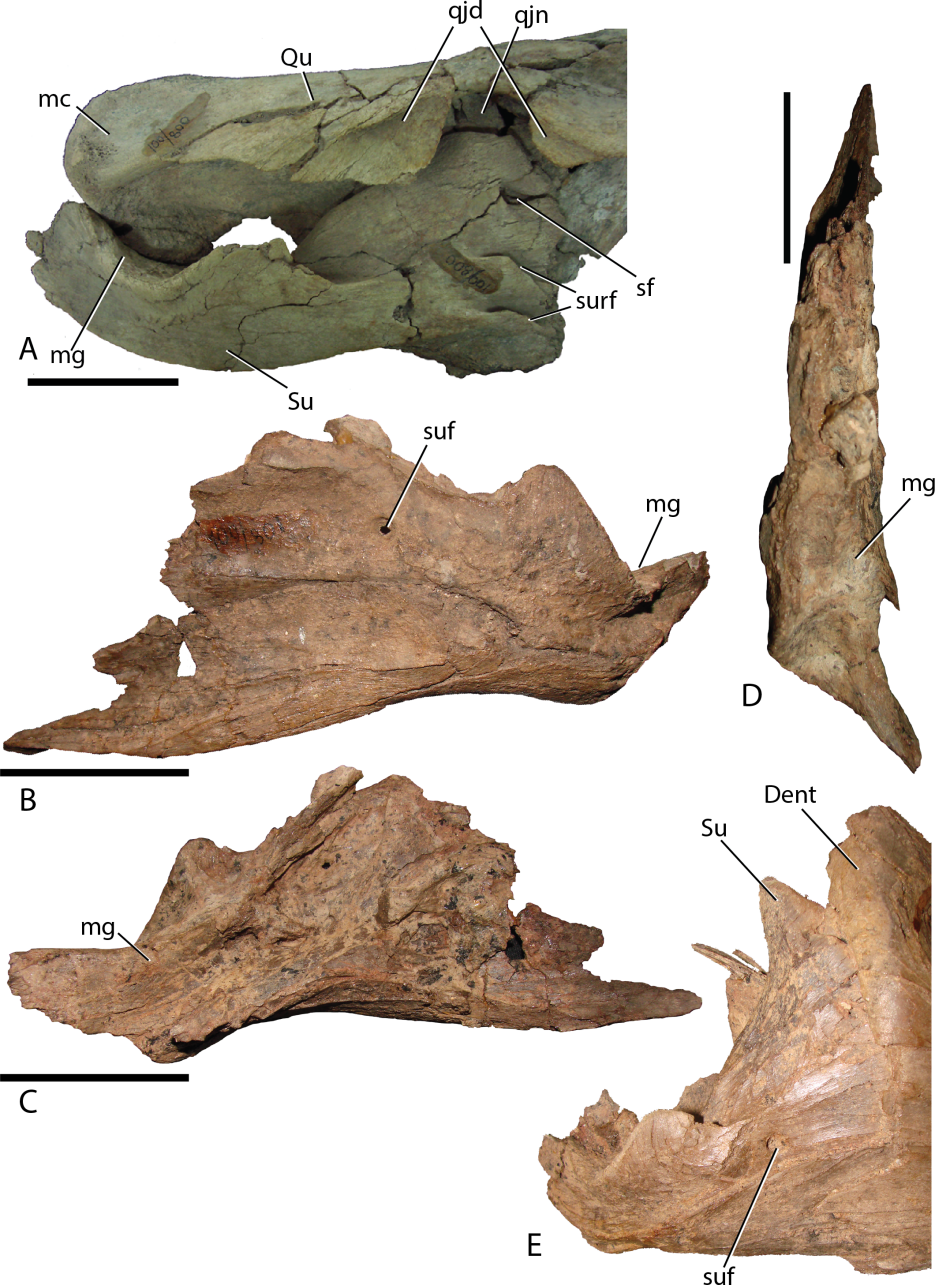
Surangular of *Choyrodon*. (A) MPC-D 100/800 right surangular and quadrate in lateral view; (B) MPC-D 100/801 left surangular in lateral view; (C) MPC-D 100/801 left surangular in medial view; (D) MPC-D 100/801 left surangular in dorsal view; (E) close-up of the articulation between the right dentary and surangular on MPC-D 100/801. Study sites: Dent, dentary; mc, mandibular condyle; mg, mandibular glenoid; qjd, quadratojugal depression; qjn, quadratojugal notch; Qu, quadrate; sf, second foramen; Su, surangular; suf, surangular foramen; surf, surangular folds. Scale bars equal five cm. Photograph credit: Terry Gates.

Surangular—This element is similar to other basal iguanodontians in that it is dorsoventrally tall and mediolaterally compressed anteriorly, broadening and shortening posteriorly to accept the mandibular condyles of the quadrate. MPC-D 100/801 clearly displays a substantial contribution to the coronoid process (Fig. 16A), whereas the preserved morphology of MPC-D 100/800 does not seem to contribute as much, possibly due to breakage (Fig. 17). The surangular foramen is clearly visible at the base of the element. A larger second foramen is positioned at the anterodorsal corner of the dentary suture. Instead of being enclosed, the latter foramen is open to the anterior surangular margin by a constricted crevasse, a condition shared with *Altirhinus* (Norman, 1998; mentioned by Norman, 1998 as an autapomorphy) and according to Barrett et al. (2009) also seen on *Lanzhousaurus* and *Jinzhousaurus*. Ventral to the second foramen are two autapomorphic folds, or grooves (Fig. 17A). The deep laterodorsally deflected folds are visible only on MPC-D 100/800, not on MPC-D 100/801, likely because the right surangular is in articulation with the dentary and this region of the bone is not preserved in the left surangular. We approximate the position of the folds as being directly ventral to the second foramen, yet it is unclear where the second foramen is preserved, if at all on MPC-D 100/801. Such an odd feature being present on only a single bone within the current hypodigm leaves room for other explanations such as individual variation. Until new evidence arises we consider this feature an autapomorphy. Angular articulation is sinuous throughout the entire ventral margin.

Angular—The angular is large and laterally facing as is common to basal iguanodontians (Norman, 2004). It articulates with the surangular by means of a long sinuous suture that extends from the caudoventral margin of the surangular. The angular angles dorsally to broadly contact the caudoventral margin of the dentary, allowing a caudal process of the dentary to overlap a depression on the angular. In lateral view, the angular contacts about one-third of the posteroventral length of the dentary (Figs. 2 and 12A).

Dentition—Maxillary dentition have a maximum width of 16.6 mm, with two teeth incorporated in the grinding surface. Each maxillary tooth has one asymmetrical main carina, with accessory ridges anterior to the main ridge (Fig. 16C) and denticles on the occlusal margin of uneroded crowns (Fig. 16D). Dentary teeth (Fig. 16E) have a maximum width of 21.8 mm with a large median carina (although smaller than the carina on the maxillary teeth) and exhibit secondary ridges on either side and in some rare cases, smaller tertiary ridges. There are large denticles on the teeth. All of the carina bend slightly distally. This configuration coincides with that of *Altirhinus* (Fig. 5B).

### Postcranial skeleton

**Figure 18:**
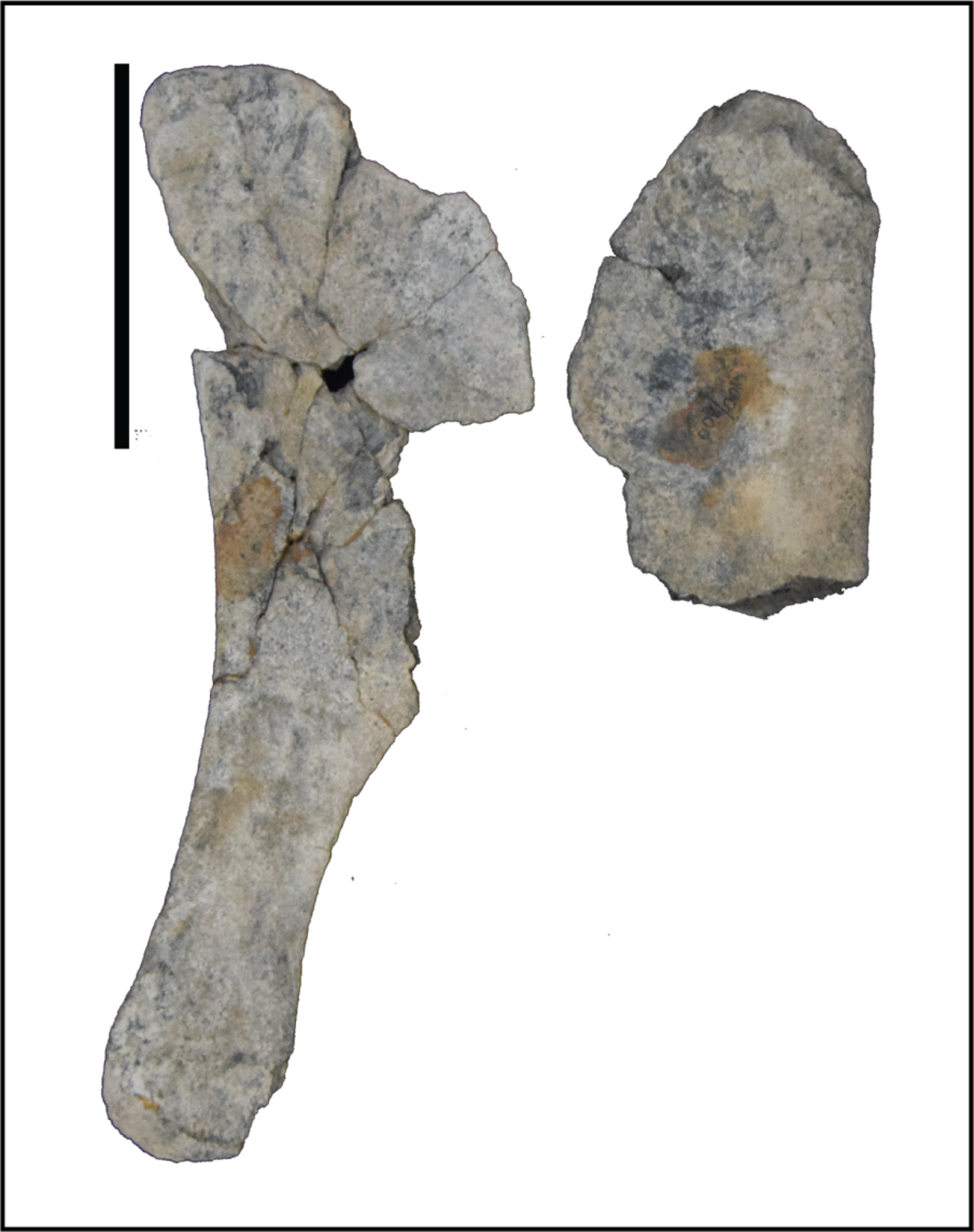
Left sternal plate of *Choyrodon* specimen MPC-D 100/800. Scale bar equals 10 cm. Photograph credit: Khishigjav Tsogtbaatar.

Sternal—The left sternal of MPC-D 100/800 (Fig. 18) consists of a slightly broad head laterally that gives way to a thin medial process, which is broken on this specimen. However, it is clear that the medial process is anteroposteriorly long, occupying at least 50% of the sternal length. The short shaft continues posteriorly to form a flat rounded tip. Lengthening of the sternal shaft in relation to the broad central process is a trait observed throughout the evolution of iguanodontians.

**Figure 19:**
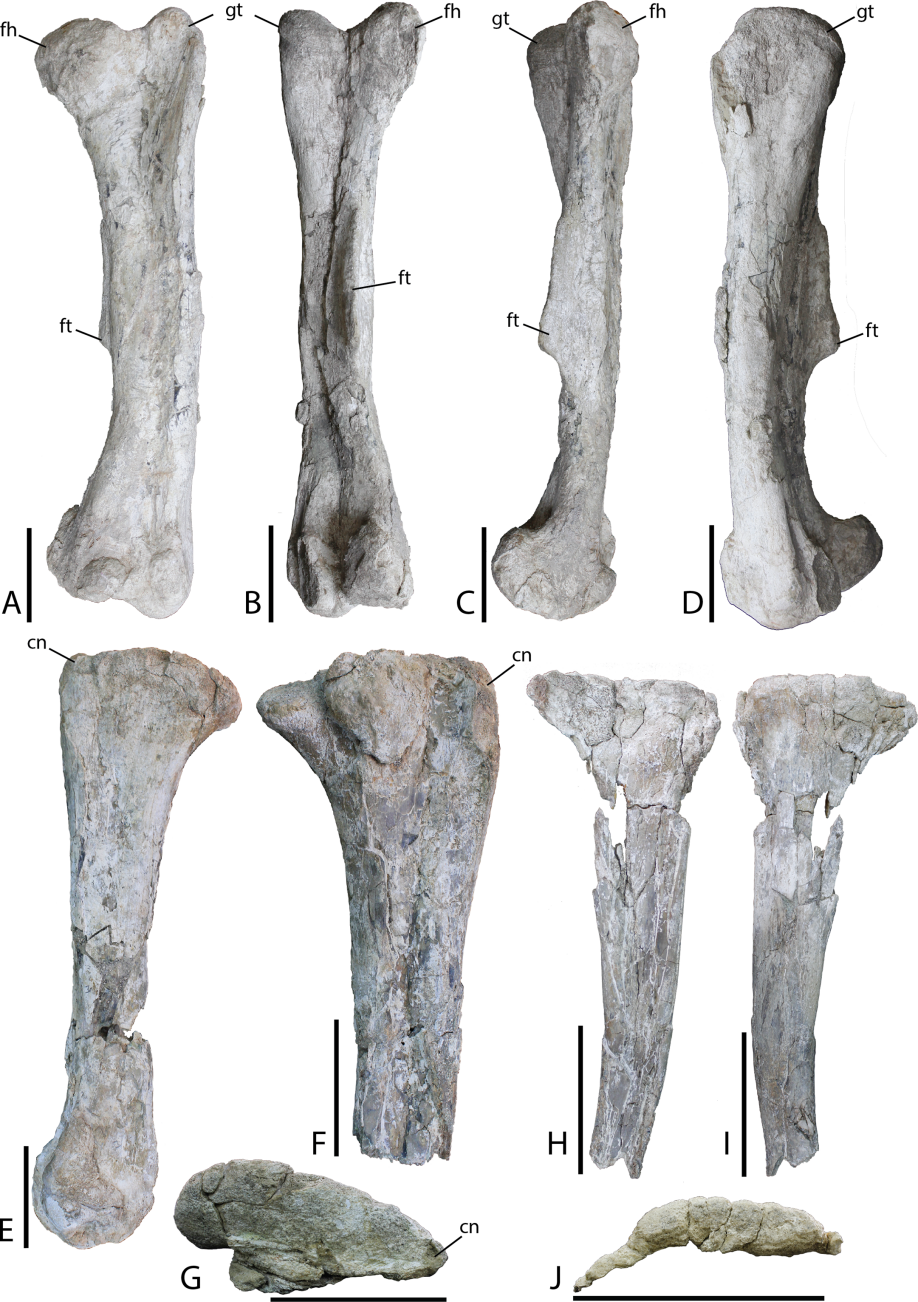
Hindlimb material from *Choyrodon* specimen MPC-D 100/803. (A) left femur in anterior view; (B) left femur in posterior view; (C) left femur in medial view; (D) left femur in lateral view; (E) right tibia in medial view; (F) right tibia in lateral view; (G) right tibia in proximal articular view (note that the lateral condyle was removed from this photograph for conservation); (H) right fibula in medial view; (I) right fibula in lateral view; (J) right fibula in proximal articular view. Study sites: cn, cnemial crest; fh, femoral head; ft, fourth trochanter; gt, greater trochanter. Scale bars equal 10 cm. Photograph credit: Tsogtbaatar Chinzorig.

Femur—The femur of MPC-D 100/803 (total length 62.6 cm estimated from photograph; Figs. 19A and 19B) exhibits a distinct head. The lesser trochanter is eroded from the element so it is impossible to detect fusion between that feature and the greater trochanter. The shaft is straight between the head and the broad and triangular fourth trochanter, whereas the distal half curves slightly laterally, although this could be a result of post-fossilization deformation. The fourth trochanter is shaped like a long low triangle, highly asymmetrical, nearly forming right triangle with the short side of the triangle located just distal the midpoint of the femur. This feature differs from *Eolambia* (trochanter located on the lower half of the element (McDonald et al., 2012a), *Probactrosaurus* (trochanter located slightly dorsal to the midpoint (Norman, 2002)). Norman (1998) mentioned a femur for *Altirhinus*, although it is apparently too fragmentary to be described (even though the lesser trochanter was part of the diagnosis). A U-shaped intercondylar groove is observed on the tibial articular surface. Lateral and medial distal condyles are equal in size.

Tibia—Slight crushing of the tibia of *Choyrodon* (MPC-D 100/803; total length estimated 59.5 cm from photograph) (Figs. 19C and 19D) obscures some features, but it appears that the medial proximal condyle is subequal in size to the lateral proximal condyle. The cnemial crest shows a more primitive feature (compared to hadrosaurids) by extending more anteriorly than laterally (e.g., see the near posterior orientation of the cnemial crest on *Edmontosaurus regalis*, Campione, 2014, fig. 13.21F). There also seems to be little to no development of the narrow wing that wraps the fibular head in more derived taxa. Following the minimal development of the cnemial crest proximally, this feature is restricted to the upper one-third of the tibial shaft. Distally, the lateral condyle is more mediolaterally compressed than its counterpart. The groove separating the two distal condyles is offset laterally and the lateral condyle possesses a sharp medial edge. The medial condyle exhibits the opposite morphology, being expanded and bulbous (Fig. 19D is the same element as Fig. 19C).

Fibula—Preserved portions of the left fibula of *Choyrodon* (MPC-D 100/803; proximal breadth across head 12.6 cm estimated from photograph) (Fig. 19E) display no remarkable anatomy, being similar to other iguanodontians.

**Figure 20:**
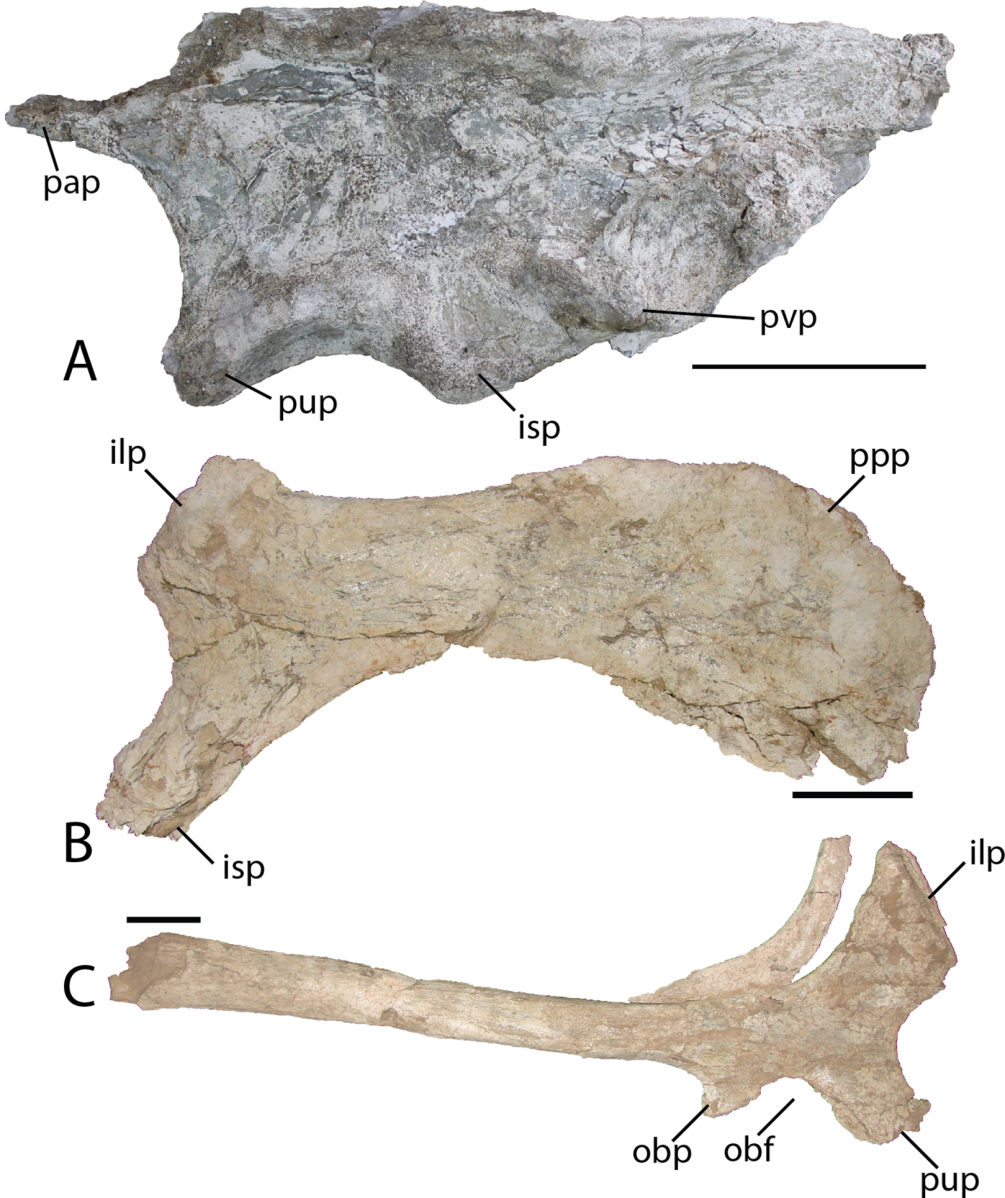
Pelvic elements of *Choyrodon*, MPC-D 100/803. (A) left ilium in lateral view; (B) right pubis in lateral view; and (C) right ischium in lateral view. Study sites: ilp, iliac process; isp, ischial process; obf, obterator foramen; obp, obterator process; ppp, prepubic process; pup, pubic process; pvp, posteroventral protuberance. Scale bars equal 10 cm. Photograph credit: Tsogtbaatar Chinzorig.

Ilium—Only the iliac plate and a small portion of the preacetabular process are present on the ilium of *Choyrodon* (MPC-D 100/803; depth of iliac plate from base of ischial peduncle to the dorsal border of the plate 16.0 cm estimated from photograph) (Fig. 20A). The general morphology corresponds to that of other basal iguanodontians having a broad iliac plate. The preacetabular process is not preserved on any specimen of *Choyrodon*. The pubic process is anteroposteriorly elongated and mediolaterally compressed, as is the ischial peduncle (MPC-D100/803; distance from base of pubic peduncle to base of ischial peduncle 10.7 cm estimated from photograph). A large protuberance is present posterodorsally to the ischial peduncle, on the lateral side of the ilium (pvp of Fig. 20A). A large protuberance such as this is not reported on *Altirhinus* (Norman, 1998; although may be present on fig. 32), *Eolambia* (McDonald et al., 2017), *Proa* (McDonald et al., 2012b), or *Probactrosaurus* (Norman, 2002), but is more often seen on derived iguanodontians such as hadrosaurids (sensu phylogenetic matrix of Prieto-Marquez, Erickson & Ebersole, 2016). On the dorsal margin of the iliac plate, the supra-acetabular process is long, originating dorsal to the acetabulum and extending posteriorly past the ischium process. Throughout its length the supra-acetabular process extends laterally, exhibiting the greatest overhang at its termination, but still exhibiting a small overhang across this distance. This overhang is similar to that seen on *Eolambia* (McDonald et al., 2012a), yet small compared to hadrosaurids (Prieto-Márquez, 2007). Campione (2014) noted that the supra-acetabular process increases in size through ontogeny within *Edmontosaurus*. A portion of the postacetabular process is present, showing no evidence of a brevis shelf. Overall, the dorsal margin of the ilium is horizontal, not exhibiting the sinuous nature of many other iguanodontians including that described for a smaller ilium of *Altirhinus* described by Norman (1998; part of the diagnosis for this taxon). Also, McDonald et al. (2017, fig. 9) showed three ilia of *Eolambia* that differ in the angulation of the preacetabular process, yet the dorsal margin of each ilium is remarkably consistent across all specimens. If this pattern continues across iguanodontians then the difference in dorsal iliac margin morphology between *Altirhinus* and *Choyrodon* might be a species-level difference in morphology as opposed to individual variation.

Pubis—The anterior process of the left pubis of *Choyrodon* (MPC-D 100/803; Fig. 20B) is more derived than that of *Proa* (McDonald et al., 2012b) and *Jinzhousaurus* (Wang et al., 2010) which are simply slightly expanded rods, in being dorsoventrally expanded and downturned distally. Other species such as *Eolambia* (McDonald et al., 2012a, 2017), *Altirhinus* (Norman, 1998), and some hadrosaurids (Prieto-Márquez, 2011) have a dorsoventrally expanded anterior pubic process that is quite similar to *Choyrodon*.

Ischium—The ischium of *Choyrodon* (MPC-D 100/803) (Fig. 20C) is morphologically similar to that of other iguanodontian taxa. The iliac process is large, being broader than the pubic shaft and creating a narrower acetabulum than *Eolambia* (McDonald et al., 2012a). *Choyrodon* has an iliac peduncle ratio less than 1:2, having both a long anteroposterior length and relatively short dorsoventral length. The shaft is straight as in *Altirhinus* (Norman, 1998), expanding distally, yet missing the distal-most end, therefore, it is unclear if a boot was present. Norman (1998) proposed a straight shaft with an axial twist as an autapomorphy of *Altirhinus* (note that *Brachylophosaurus* is described as having a straight ischial shaft with an axial twist; Prieto-Márquez, 2007). *Choyrodon* (MPC-D 100/803) seems to lack the axial twist and also appears to expand slightly distally. Straight ischial shafts are not a common feature among basal iguanodontians (although hadrosauroids and saurolophine hadrosaurids seem to possess this feature more commonly, e.g., Brett-Surman & Wagner, 2007; Prieto-Márquez, 2007; Gates et al., 2014) and this may be in important phylogenetic link between the two iguandontians from the Khuren Dukh Formation.

Caudal vertebra—An isolated midcaudal vertebra from *Choyrodon* (MPC-D 100/800) possesses a nearly triangular centrum in anterior view. Transverse processes arise from the apex of the centrum.

## Paleohistlogy

**Figure 21:**
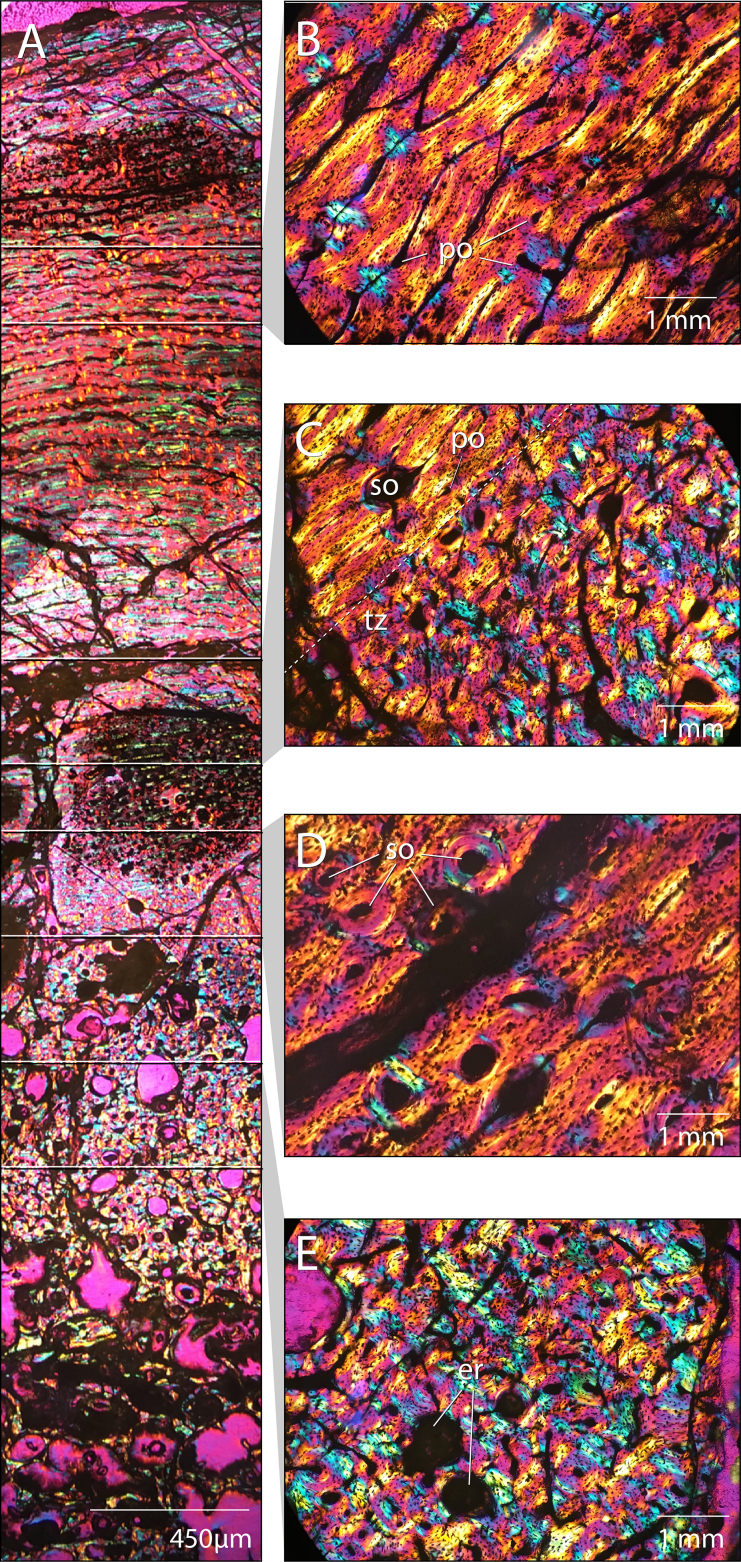
Femoral paleohistology of *Choyrodon* MPC-D100/803. (A) complete section from the periosteal surface (top) to approximate center of medulla (bottom) with sections delimited showing areas of close-up bone microstructure; (B) outer cortex composed of more organized woven-fibered tissue with plexiform to laminar vascularity; (C) transition zone between highly disorganized, woven-fibered bone tissue with reticular vascularization and a the more organized woven-fibered tissue with plexiform to laminar vascularization; (D) mid-cortex with secondary osteons (Haversian systems); (E) inner cortex bearing erosion rooms. All sections under polarized light; scale bars as noted on subsections. Images captured on a Nikon Eclipse Ci-Pol microscope. Study sites: er, erosion rooms; po, primary osteon; so, secondary osteon (Haversian system); tz, transition zone. Photograph credit: Lindsay Zanno.

As a means of independently assessing skeletal maturity of MPC-D 100/803 at the time of death, we generated paleohistological thin sections of the tibia and femur (Fig. 21). Sections were taken midshaft and processed at the Institute of Paleontology and Geology, Mongolian Academy of Sciences. Both elements are heavily crushed rendering it impossible to trace specific features throughout the entire circumference of the long bones and microbial/and or fungal invasion has obscured portions of histological structure throughout. Here we describe and figure only the femoral section, which is better preserved.

The cortex is composed of fibrolamellar bone tissue (Fig. 21A). The inner cortex is highly disorganized and consists of a woven-fibered matrix with reticular vascularization, dense primary osteons and large erosion rooms (Fig. 21E). This tissue transitions sharply in the middle cortex (Fig. 21C) to a more organized woven-fibered tissue with variably plexiform and laminar vasculature and reduced vascularization, comprising the peripheral half of the cortical section (Fig. 21B). This transition suggests a slowing of growth rate in the outer cortex (Francillon-Viellot et al., 1990; De Margerie et al., 2004). Secondary osteons (Haversian canals) are observed (Fig. 21D), which along with erosion rooms (some bearing lamellar tissue along the margins), indicate secondary remodeling of primary bone tissue. Secondary osteons are restricted to the area just superficial to the transition zone (Figs. 21C and 21D) and have not extensively invaded the cortex; no cross-cutting of Haversion systems is observed. No lines of arrested growth or annuli aside from the single transition of tissue type from rapid to slower deposition midcortex are observed. However, it is possible other indicators are present and cannot be identified due to extensive crushing and cracking of the section, since growth markers are observed in other iguanodontians (Werning, 2012; McDonald et al., 2017; Horner et al., 2009; Hübner, 2012; Stein et al., 2017). Overall, the bone tissue remains well vascularized in the outermost cortex and no external fundamental system (EFS) is visible.

We compared the femoral histology of MPC-D 100/803 to well-documented femoral growth stages from other ornithopod taxa including *Dryosaurus*, *Tenontosaurus*, and *Dysalotosaurus* (Horner et al., 2009; Hübner, 2012; Werning, 2012). The combination of an absence of an EFS, presence of plexiform/laminar organization of tissues in the mid to outer cortex, and secondary remodeling of the inner cortex is observed only in subadult growth stages and suggests that this individual was a young subadult and still growing at the time of death. This finding compares well with patterns of sutural closure on *Choyrodon*. Furthermore, sutural fusion is observed on the frontals, postorbitals, and parietals, yet not the braincase, suggesting MPC-D 100/800 and 100/801 were still actively growing, but beginning to approach skeletal maturity at the time of death.

## Phylogenetic Analysis

We tested the phylogenetic relationships of *Choyrodon* using two independent character matrices: Wenhao & Godefroit (2012) (108 characters and 26 taxa) and Norman (2015) (105 characters and 28 taxa). Within the Norman (2015) matrix we recoded character states for several taxa in character 63 (dentary teeth, primary ridge: absent (0), mesial/median position and prominent (1), distally offset and modestly developed (2)). In this case the taxa *Parasaurolophus walkeri*, *E. regalis*, and *Saurolophus osborni* were all coded as state 3 in the published matrix when no such state exists for that character as currently written; instead we changed the coding to state 1. A nexus file for each analysis can be found in the Supplementary Material. Phylogenetic analyses were performed in PAUP v. 4.0a159 (Swofford, 2002) using heuristic searches both for the Wenhao & Godefroit (2012) matrix and for the Norman (2015) matrix; in both cases, all search options remained at default settings, except that we retained 10 trees per stepwise addition as opposed to the default of one tree per stepwise addition. Bootstrap calculations of 100 replicates were performed using default settings except that stepwise additions were randomly generated. Bremer supports, also conducted in PAUP v. 4.0a159, used the same heuristic search options as the original tree search. We conducted analyses with character ordering following Wenhao & Godefroit (2012) as well as analyses where all characters were treated as unordered as a null hypothesis without injecting a priori structure into the evolution of traits. Within the Norman (2015) matrix all characters were treated as unordered by us and the original author (Table 1).

**Table 1:** Choyrodon phylogenetic statistics.

Analysis	MPT’s	Tree length	CI	RI	RC
Wenhao & Godefroit (2012) matrix					
Ordered as published	6	246	0.589	0.799	0.471
Unordered	3	242	0.599	0.788	0.474
Unordered recoded	87	242	0.599	0.788	0.472
Norman (2015) matrix					
Original coding	27	326	0.552	0.766	0.423
Recoded	3	325	0.554	0.767	0.425

One anatomical difference between *Choyrodon* and *Altirhinus* is the presence of an open antorbital fenestra in the former species. The size and position of the antorbital fenestra is known to vary ontogenetically in other iguanodontians (Carpenter, 1999; Verdu et al., 2015) and closure of the antorbital fenestra during ontogeny has been proposed as a hypothetical step between species having an antorbital fenestra at adulthood versus those without (Witmer, 1997). The growth stage of the holotype specimen of *Altirhinus* is unknown, however, our results suggest a subadult stage for specimens of *Choyrodon*. Therefore, we explored the impact of ontogenetic closure of the antorbital fenestra on the phylogenetic relationships of *Choyrodon* in an attempt to test the hypothesis that these specimens are conspecific. For this, we reran the phylogenetic analyses after recoding all characters on *Choyrodon* associated with the antorbital fenestra to reflect the condition seen in *Altirhinus*, in which the antorbital fenestra is closed to the exterior (character 26 to state 2 for Wenhao & Godefroit (2012) and Norman (2015) character 10 to state 3 and character 11 to state 1), and explored the impact of this change on our results.

**Figure 22:**
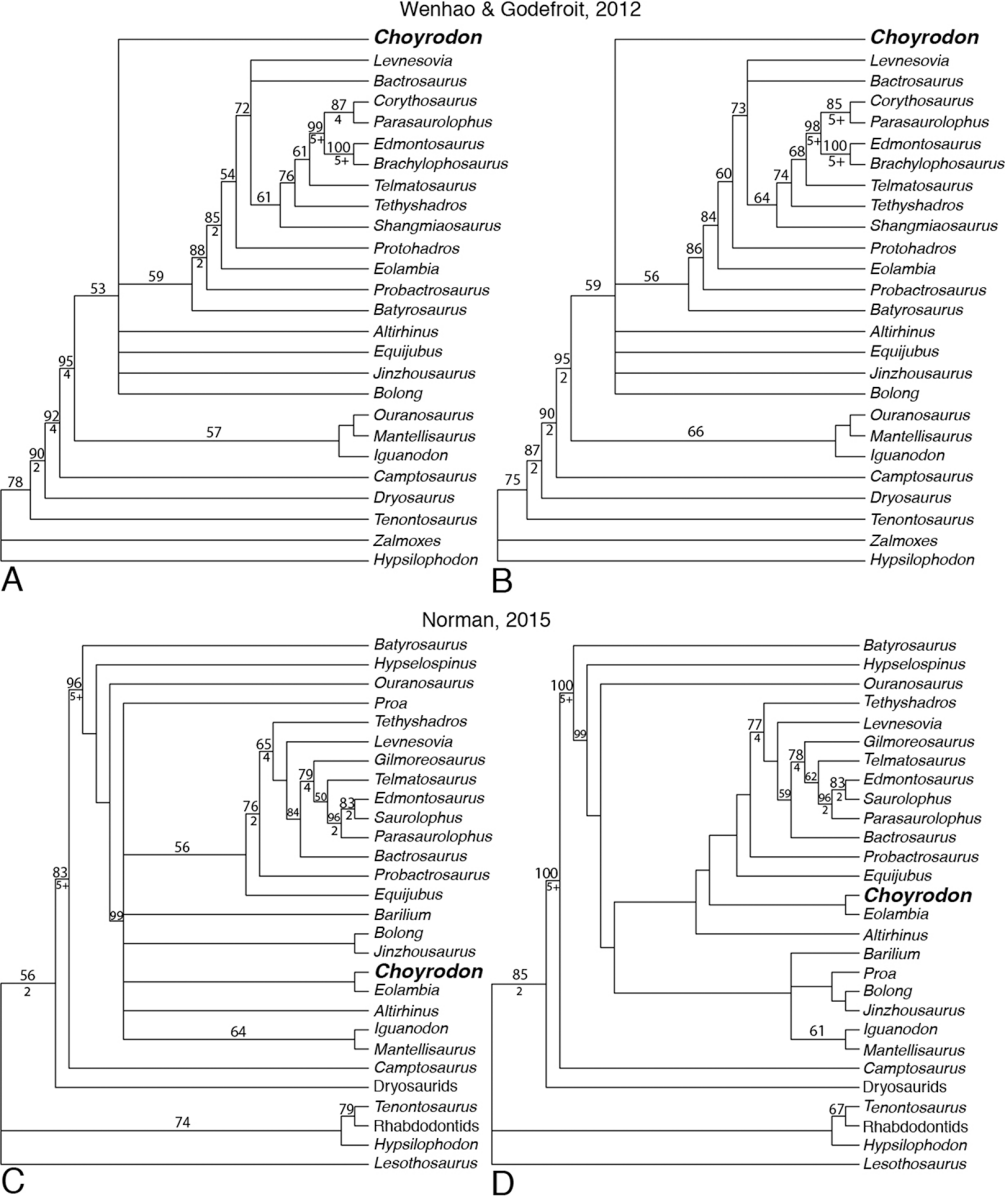
Phylogenetic trees showing relationships of iguanodontians including. *Choyrodon barsboldi* Wenhao & Godefroit (2012) (A) strict consensus of six most parsimonious trees running matrix with character ordering as suggested by authors; (B) strict consensus of 87 most parsimonious trees running matrix with all characters unordered and with *Choyrodon* coded with an open antorbital fenestra. Norman (2015) (C) strict consensus of 27 most parsimonious trees; (D) strict consensus of the three most parsimonious trees with *Choyrodon* coded as if the antorbital fenestra is closed externally. Numbers above branches are the bootstrap values shown only for those greater than 50%. Bremer support values are shown below branches for those that have a score greater than 1. See Table 1 for phylogenetic tree scores.

Strict consensus of trees resulting from the Wenhao & Godefroit (2012) matrix with both ordered (Fig. 22A) and unordered (Fig. 22B) characters produces a large polytomy including *Choyrodon*, *Bolong*, *Jinzhousaurus*, *Equijubus*, *Altirhinus*, and a clade containing more specialized hadrosauroids. Minimum tree length was lower when characters were considered unordered. Coding *Choyrodon* as having a closed antorbital fenestra did not impact this result.

Greater resolution was obtained from the Norman (2015) dataset. Strict consensus suggests a sister-taxon relationship with the North American Cenomanian taxon *Eolambia* when *Choyrodon* is coded as possessing either an open (Fig. 22C) or closed (Fig. 22D) antorbital fenestra. Unambiguous synapomorphies between *Choyrodon* and *Eolambia* include character 1, 64, and 67, which corresponds to a trapezoidal shaped occiput, a dominant median ridge with secondary ridges on dentary teeth, and maxillary teeth lanceolate and equal in size to opposing dentary teeth. Ambiguous synapomorphies include numbers 8, 36, 49, 80, and 99. Together these taxa are unresolved with respect to iguanodontians (*Iguanodon* + *Mantellisaurus*), other early diverging hadrosauroids (*Altirhinus*, *Barilium*, *Proa*, (*Bolong* + *Jinzhousaurus*), and more specialized hadrosauroids (*Equijubus* +) when *Choyrodon* is coded as possessing an antorbital fenestra (Fig. 22C). However, under a hypothesized scenario in which the antorbital fenestra of subadult *Choyrodon* closes at skeletal maturity, *Altirhinus* is recovered as having diverged earlier than a clade containing *Choyrodon* + *Eolambia* and all more specialized hadrosauroids, whereas, *Barilium*, *Proa*, *Bolong*, *Jinzhousaurus*, *Iguanodon*, *Mantellisaurus* are recovered as a distinct clade of iguanodontians (Fig. 22D).

## Discussion

### *Choyrodon*–*Altirhinus* character conflict

The *Altirhinus* holotype specimen (PIN 3386) lacks the skull roof and braincase, and no histological study has yet been completed, so aside from absolute size differences comparison of maturity between specimens of *Choyrodon* and *Altirhinus* cannot be made—no vertebrae are present in the *Altirhinus* or *Choyrodon* type specimens (sensu Norman, 1998). However, given the co-occurrence of the iguanodontians *Choyrodon* and *Altirhinus* in the Khuren Dukh Formation, we evaluate the possibility that observed differences between these taxa are attributable to ontogeny rather than species-specific morphology.

Ornithopod skulls are documented to undergo significant change throughout ontogeny, with lambeosaurine hadrosaurids exhibiting the most extreme ontogenetic modifications (Evans & Reisz, 2007; Farke et al., 2013). Even among those species exhibiting the most ontogenetic change, certain regions of the skull modify at different rates. Elements such as the quadrate remain relatively static, whereas the masticatory apparatus tends to reflect observable lengthening of the jaws and downturning of the dentary (e.g., *Corythosaurus*, Evans, Forster & Reisz, 2005; *Gryposaurus* (UMNH VP 13970), Supplementary Information). Subtle changes to the jugal reflect a decrease in relative orbit size (Gates et al., 2007), and a reduction in frontal doming nearing skeletal maturity is documented in several iguanodontian taxa (Evans & Reisz, 2007; Evans, Reisz & Dupuis, 2007). As mentioned above, species of the hadrosaurid genus *Gryposaurus* document dorsal elevation of the posterior skull throughout ontogeny, as recorded in the posterior process of the postorbital as well as other bones (Farke & Herrero, 2014).

Despite sharing a similar appearance and occurring in the same stratigraphic beds, *Choyrodon* and *Altirhinus* diverge in many aspects of their morphology that we do not accept as plausibly related to ontogeny, that is, the smaller-bodied *Choyrodon* is not likely to represent a skeletally immature *Altirhinus*. Here we discuss each in turn.

1. In *Choyrodon* the quadratojugal notch of the quadrate is located more dorsally and lacks the dorsal prong seen on the ventral boundary of the quadratojugal notch of *Altirhinus* (Norman, 1998). Ontogenetic change to the quadrate of iguanodontians is minimal, at least in lambeosaurine hadrosaurids (Gates et al., 2007), and this variation cannot easily be attributable to ontogeny.
2. The posterior process of the postorbital of *Choyrodon* is steeper than that observed in *Altirhinus*. The angulation of the posterior process of the postorbital has been demonstrated to increase relative to the horizontal in saurolophine hadrosaurids during ontogeny (Farke & Herrero, 2014). Therefore the pattern observed in *Choyrodon* and *Altirhinus* is opposite what would be expected in ontogeny.
3. Several features observed on the palatine of *Choyrodon* are significantly more developed than what is observed on *Altirhinus*. For instance, the anterior process is exaggerated in *Choyrodon*, and the dorsal surface is similarly well-arched. This generally contradicts the expected pattern of exaggeration with growth observed in other dinosaurian taxa.
4. The overhang on the posterior margin of the skull of *Choyrodon* that we interpret as the opisthotic, and the folding of the anteroventral surangular are features not observed on other iguanodontians to date. We do not consider these to be features likely linked to ontogeny.
5. *Altirhinus* possesses a short, laterally deflecting, bifurcating ventral process on the predentary (Norman, 1998). The homologous feature seen on *Choyrodon* specimen MPC-D 100/801 is long, narrow, and straight (Fig. 17). It seems unlikely that the ventral process of the predentary would alter its shape to this degree during growth.
6. *Choyrodon* possesses an antorbital fenestra—a feature not observed in *Altirhinus*. Over the course of ornithopod evolution, the antorbital fenestra decreased in size externally until completely sheathed over by bone, open only internally as an antorbital cavity (Witmer, 1997). Expansion of the masticatory apparatus and the nasal complex is proposed to be the reason for the closure (Witmer, 1997). This pattern, together with the fact that the antorbital fenestra is known to reduce in size in with growth in some iguanodontian species (e.g., *Dryosaurus*; Carpenter, 1999) and close entirely in *Alligator* (although the opening in *Alligator* never actually reaches the structural finishing to be called a fenestra; sensu Witmer, 1997), raises the possibility that the presence of an antorbital fenestra in known specimens of *Choyrodon* is simply the result of its subadult growth stage and that this is not a reliable trait differentiating this species from *Altirhinus*. Although such a hypothesis (closure of the antorbital fenestra in skeletally mature individuals of *Choyrodon*) cannot currently be ruled out in light of the absence of adult individuals, we are not aware of any evidence among ornithopods for closure of the antorbital fenestra within a single species during growth.

Finally, if expansion of the nasal complex and tooth batteries in large bodied individuals is the underlying cause for closure of the antorbital fenestra in ornithopod evolution, then smaller body size in mature individuals of *Choyrodon* as opposed to *Altirhinus* might also explain retention of this feature in the former species. As comparative histology between these species is unknown, this hypothesis cannot be ruled out. Based on results from this study and others over the past 20 years the only unique trait of *Altirhinus* remaining from the original Norman (1998) description is that of “rostral tip of nasals strongly arched.”

### Phylogenetic hypotheses

The inclusion of *Choyrodon* disrupted the nearly bifurcating tree of Wenhao & Godefroit (2012) and Norman (2015), producing a large polytomy of derived non-hadrosauroid iguanodontians. This is not unexpected given that *Choyrodon* shares a variety of traits with other iguanodontian species. Under no combination of matrices, character ordering, or hypothesized ontogenetic changes in morphology did we recover an exclusive relationship between *Choyrodon* and *Altirhinus*. Further, under no combination of our analyses did we recover *Choyrodon* as a taxon having definitively diverged earlier than *Altirhinus*. Such a result is inconsistent with studies documenting that subadult growth stages resolve as earlier diverging taxa in phylogenetic studies (Campione et al., 2013; Fowler et al., 2011), and fails to support the hypothesis that *Choyrodon* is a juvenile *Altirhinus*.

Interestingly, when coded as having a closed antorbital fenestra using the Norman (2015) matrix, *Choyrodon* is recovered in a sister-taxon relationship with the North American taxon *Eolambia*, known from the Cenomanian-aged Mussentuchit Member of the Cedar Mountain Formation. Although the presence of a closed antorbital fenestra in an adult *Choyrodon* is hypothetical, a sister-taxon relationship between the early diverging, “mid”-Cretaceous hadrosauroids *Choyrodon* and *Eolambia* is consistent with a myriad of other studies recovering close evolutionary ties between Asian and North American taxa across a robust variety of vertebrate clades during this time interval (e.g., mammals, squamates, turtles, and dinosaurs, see Cifelli et al., 1997; Kirkland et al., 1999; Zanno & Makovicky, 2011, 2013).

### Taphonomy

The paleoenvironment of the lower Khuren Dukh Formation is interpreted as a series of braided streams and lakes, with the Lower Member (∼65 m thick) more specifically recording the downstream migration of longitudinal bars within a braided river channel, whereas the carbonaceous mudstones are interpreted as gradually-filled abandoned channels (Ito et al., 2006). Two sediment types are nearly ubiquitous throughout the Khuren Dukh Lower Member lithofacies association 1, which is where the majority of fossils derives: an organic-rich, silty-mudstone in the upper formation and a laterally extensive, course grained white–gray sandstone in the lower (Novodvorskaya, 1974; Ito et al., 2006). Fossil specimens that occur in the organic-rich layers are dark brown in color and typically have poor bony quality (e.g., *Harpymimus*, MPC-D 100/29). However, MPC-D 100/801 has exceptional bone quality and possesses a remarkably well-preserved series of cracks mostly likely recording prefossilization subaerial exposure and bone desiccation. Examples of radial-style and linear desiccation cracks can be seen on the MPC-D 100/801 premaxilla (Figs. 3A and 3B), nasal (Figs. 4A and 4B), lacrimal (Fig. 8), quadratojugal (Figs. 4A, 4B and 10), and the frontals (Figs. 11A and 11C). The quality of these features are found in abundance on fossils from the La Brea Tar Pits (T. Gates, 2001, personal observation) but are rarely reported from other sites (Fiorillo, 1988; Varricchio, 1995).

MPC-D 100/800 and MPC-D 100/803 have a preservational mode that differs substantially from MPC-D 100/801 despite being from the same formational member. The former two specimens were deposited in the white–gray braided river channels that contain coarse-grained arkosic sand and therefore, the bone is ashy white and etched from abrasion (all preburial broken surfaces are rounded). MPC-D 100/801 was discovered in a carbonaceous layer between successive white channel sands. These opposing depositional environments are part of the sedimentary architecture of the Khuren Dukh Formation Lower Member (Ito et al., 2006). All specimens are disarticulated suggesting that despite burial within a river channel, sediment coverage did not occur quickly, or that the Khuren Dukh channels are of a braided river system with intermittent flow as proposed by Ito et al. (2006). Fish fossils found within the river channels are of excellent preservational quality compared to the ornithopod fossils, supporting the hypothesis of subaerial weathering of the dinosaurs prior to burial.

## Conclusion

The new iguanodontian, *C. barsboldi*, from the Albian Khuren Dukh locality of the Khuren Dukh Formation of Mongolia is described here based on approximately seven autapomorphic traits found throughout the skull in addition to a unique combination of several skull traits found in other iguanodontians. Of the autapomorphic traits, some of the most notable include an apparent overlap of the opisthotic onto the supraoccipital and two osteological folds on the anteroventral surangular. *Choyrodon* shares with more basal taxa an open antorbital fenestra, yet also possesses more derived traits such as a downturned dentary and an enlarged narial fenestra. An enlarged narial fenestra is also found in, and a defining feature of, the taxon *A. kurzanovi*, which was discovered from the same formation as *Choyrodon*. Osteohistology indicates that one specimen of *Choyrodon* was a subadult individual still actively growing at the time of death. Given that all specimens are approximately the same size we assume that all specimens share a similar growth stage.

Dismissing the number of unique features characterizing *Choyrodon*, we attempted to test the hypothesis that *Choyrodon* represents a subadult growth stage of the contemporary taxon *Altirhinus* by conducting a phylogenetic analysis in which we inferred that the antorbital fenestra of *Choyrodon* would close with skeletal maturity. Hypothesized relationships between *Choyrodon* and *Altirhinus* did not change in these iterations. Although we cannot rule out the hypothesis that specimens of *Choyrodon* are subadult specimens of *Altirhinus* we find no conclusive evidence to support an assignment. Firstly, given that the growth stage of the holotype of *Altirhinus* is unknown it cannot be documented that these species are represented by different ontogenetic stages, and although specimens of both species are known from the same formation, stratigraphic placement is not a justifiable reason for inferring taxonomic identity. Moreover, we note that many of the morphological differences are not consistent with ontogenetic changes observed in other hadrosauroids. Finally, closure of the antorbital fenestra between subadult and adult growth stages is undocumented in iguanodontians to date, and when taken in sum none of our phylogenetic tests, including those incorporating the potential of antorbital fenestra closure in mature *Choyrodon*, recovered *Choyrodon* as having diverged earlier than *Altirhinus*, a finding that would be consistent with it being a younger growth stage (Campione et al., 2013; Fowler et al., 2011). Thus, we find the weight of evidence currently supports the hypothesis that *Choyrodon* is a distinct taxon. However, we acknowledge that without definitively overlapping ontogenetic stages for comparison, an ontogenetic hypothesis cannot be disproved. Until such discoveries come to light we find it most conservative to follow the current morphological and phylogenetic evidence in considering these taxa as distinct species.

Taphonomically, the holotype specimen of *Choyrodon* (MPC-D 100/801) displays incredibly well-preserved, weathering crack marks caused from subaerial exposure—one of the best examples of this taphonomic modification yet reported on a dinosaur fossil. Given the interpretation of the paleoenvironment as an organic-rich, wet riparian system, the bone condition seems to contrast with assumptions of taphonomic potential where such a humid system would rot instead of crack (Scherzer & Varricchio, 2010). Although Behrensmeyer (1978) noted that microenvironments are what drives bone decay since there is not a close association between the overarching environment a bone lies in and the patterns of degradation. More research on modern weathering regimes is required to more fully understand the implications of this phenomenon.

## Supplemental Information

10.7717/peerj.5300/supp-1 Supplemental Information 1 Phylogenetic matrix of Wenhao and Godefroit unordered.

10.7717/peerj.5300/supp-2 Supplemental Information 2 Phylogenetic matrix of Wu and Godefroit ordered.

10.7717/peerj.5300/supp-3 Supplemental Information 3 Phylogenetic matrix of Wu and Godefroit recoded and unordered.

10.7717/peerj.5300/supp-4 Supplemental Information 4 Phylogenetic matrix of Norman recoded.

10.7717/peerj.5300/supp-5 Supplemental Information 5 Phylogenetic matrix of Norman.

10.7717/peerj.5300/supp-6 Supplemental Information 6 Image of Gryposaurus sp. dentary (UMNH VP 13970 of juvenile individual from the Campanian-aged Kaiparowits Formation of Utah. Lateral view of dentary showing near horizontal ventral margin, which contrasts with the more down-turned dentary of adults (e.g., Gates & Sampson, 2007).

## Institutional Abbreviations

UMNH VP: Natural History Museum of Utah, Salt Lake City, UT, USA
MPC-D: Institute of Paleontology and Geology, Mongolian Academy of Sciences, Mongolia.

## Additional Information and Declarations

### Data Availability

The following information was supplied regarding data availability: The phylogenetic matrices are provided as nexus files in Supplementary Files. These contain all characters and codings used in our analyses.

### New Species Registration

The following information was supplied regarding the registration of a newly described species: Publication LSID: urn:lsid:zoobank.org:pub:E44CAFD0-8E9A-4025-80F2-78011BA0594F. IGUANODONTIA (Dollo, 1888) (sensu Sereno, 1986): urn:lsid:zoobank.org:act:07C25E64-6C15-40C3-8A61-A6378D8494AD. Choyrodon gen. nov.: urn:lsid:zoobank.org:act:A11FF4A2-903D-4BE5-84A2-66D19E545A61. *Choyrodon barsboldi* sp. nov. urn:lsid:zoobank.org:act:92591A61-54AD-4E74-B47F-2F1135305E9A.
