# Supplementary figures and images for: A new iguanodontian (Dinosauria: Ornithopoda) from the Early Cretaceous of Mongolia
Source: PeerJ. 2018 Aug 3;6:e5300. doi: 10.7717/peerj.5300 (PMC6078070; doi:10.7717/peerj.5300)

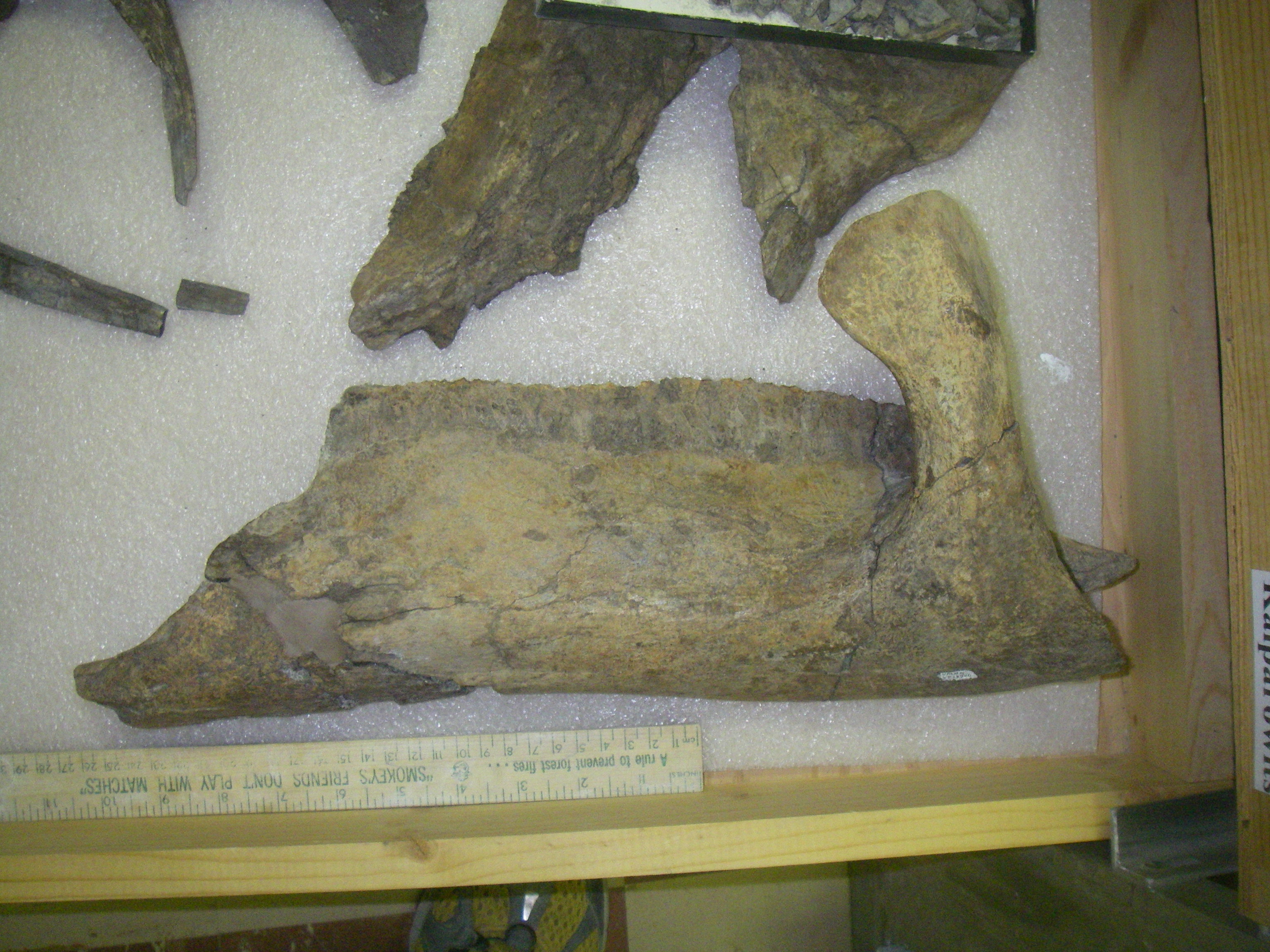

Supplement: Supplemental Information 6 — Lateral view of dentary showing near horizontal ventral margin, which contrasts with the more down-turned dentary of adults (e.g., Gates & Sampson, 2007). [file peerj-06-5300-s006.jpg]
